# Mining hydroformylation in complex reaction network *via* graph theory†

**DOI:** 10.1039/d1ra03395f

**Published:** 2021-07-01

**Authors:** Keisuke Takahashi, Maeda Satoshi

**Affiliations:** Department of Chemistry, Hokkaido University North 10, West 8 Sapporo 060-8510 Japan keisuke.takahashi@sci.hokudai.ac.jp; Institute for Chemical Reaction Design and Discovery (WPI-ICReDD), Hokkaido University Kita 21 Nishi 10, Kita-ku Sapporo Hokkaido 001-0021 Japan smaeda@eis.hokudai.ac.jp

## Abstract

Data science is introduced to identify the reactant, product, and reaction path in the chemical reaction network. Cobalt catalyzed hydroformylation is investigated where the reaction network is built *via* first principles calculations. The closeness centrality and high frequency node are found to be the reactant cobalt tetracarbonyl hydride. In addition, betweenness centrality uncovers three reaction paths which have the products of aldehyde, CH_2_O, and CO_2_, respectively. The energy profile determines that the reaction path leading to aldehyde is energetically favored; thus, the reaction path for cobalt catalyzed hydroformylation is identified without kinetics. Hence, the proposed approach can act as a first step towards understanding the complex chemical reaction network and towards further kinetic understanding of the chemical reaction.

## Abstract 2

Data science is introduced to identify the reactant, product, and reaction path in the chemical reaction network.

## Graphical abstract

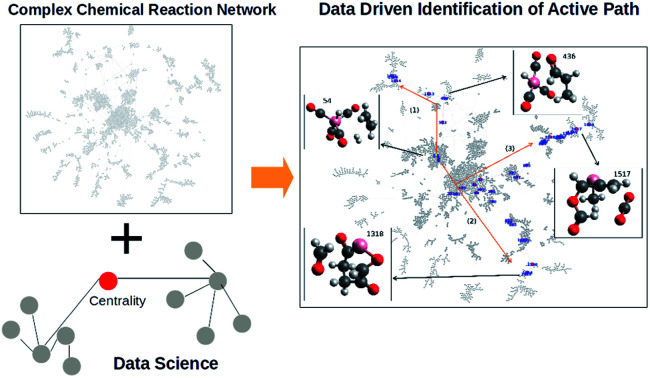

## Introduction

Identifying the reaction path within a chemical reaction is a challenging task as a chemical reaction involves complex molecular interactions. For such situations, the introduction of first principles calculations gives insight towards the atomic level understanding of molecular interactions. In particular, the potential energy surface generated by first principles calculations elicits the details of the molecular interactions on an atomic scale.^1,2^ This essentially allows for the generation of a chemical reaction network in terms of molecular interactions.^3–7^ In general, chemical kinetics is coupled with a calculated chemical reaction network in order to determine the reaction pathway.^8,9^ However, one can consider that hidden trends and patterns for identifying the reaction path within the chemical reaction network should be present, considering that the energy landscape created by first principles calculation follows certain rules. In view of how a chemical reaction network is formed, the network can be treated as a graph data structure.^5,10^ Additionally, it is reported that graph theory can be used in order to extract knowledge from a chemical network.^11^ Here, data science, particularly graph theory, is implemented in order to search the reaction paths in a chemical reaction network.

Hydroformylation is selected as the prototype reaction where the reaction involves the production of aldehydes from alkenes.^12,13^ In particular, cobalt catalyzed hydroformylation is investigated for considering homogeneous catalysis as the details of the reaction process are rather complex.^8,14,15^ The chemical reaction network of cobalt catalyzed hydroformylation is constructed *via* first principles calculations where the atomic interactions of CO dissociated cobalt tetracarbonyl hydride HCo(CO)_3_ with ethylene (C_2_H_4_), hydrogen H_2_, and carbon monooxide (CO) are considered. Reaction paths in the chemical reaction network for cobalt catalyzed hydroformylation are sought for *via* data driven analysis based on graph theory.

## Methods

### Computational method

The chemical reaction path network is explored using the artificial force induced reaction (AFIR) method combined with first principles calculations.^16^ AFIR induces chemical transformations by applying force and finds reaction paths based on the force-induced paths. The search is performed using the single-component algorithm of AFIR (SC-AFIR) starting from 200 initial structures produced by generating mutual positions and orientations among HCo(CO)_3_, CO, C_2_H_4_, and H_2_ randomly. Additionally, the model collision energy parameter *γ* of the AFIR method is set to 300 kJ mol^−1^, where *γ* defines an approximate upper limit of the barrier the artificial force can eliminate. During the search, additional weak force with *γ* = 0.65 kJ mol^−1^ is applied to all atom pairs in the system in order for the molecules to not separate too far in this system. All AFIR paths were reoptimized using the path optimization method using the locally updated planes (LUP) method, where the network of LUP paths are discussed below.^17^ The traffic volume is an index showing the total amount of population influx to and outflux from each local minimum within the simulation time *t*_MAX_. Therefore, local minima having large traffic volume values are regarded to be kinetically important. The traffic volume *Λ*_i_ is computed for all local minimum structures, and paths are searched preferentially from those having large values.^9^ For the traffic volume calculations, the initial population is evenly distributed to local minimum structures having the same bond connectivity to the initial species where the reaction time *t*_MAX_ was set to 3600 seconds, the reaction temperature set to 300, 400, and 500 K, and the model temperature parameter *T*_R_ set to 4000 K.^9^ The search is terminated when the latest *N* successful paths do not update the structural types of the top *M* traffic volumes, where *N* and *M* were set to ten and three times, respectively, of the total number of atoms in the system, a structural type stands for a group of local minimum structures having the same bond connectivity pattern and a successful path corresponds to a path connecting different structural types. All electronic structure calculations are done by the Gaussian 16 program where the ωB97X-D functional and LanL2DZ basis set are implemented.^18^ All structural displacements were taken by a development version of the GRRM program (version on April 9th, 2020).^19^ Note that the generated data is a preliminary study of the chemical reaction created for data science applications and requires further study for a more, detailed understanding of the energetics of the chemical reaction. Details of the SC-AFIR method and the traffic volume index are described in previous work.^9,16^

### Data science method

The chemical reaction network for cobalt catalyzed hydroformylation is investigated using data science and graph theory. The created reaction network is transformed into a directed graph where source and target nodes are defined as reactants and products, respectively. The activation energy barrier is represented as node edges and is reflected in edge weight. Gephi is then implemented for graph visualization and analysis.^20,21^ Force Atlas 2 is used for graph visualization while closeness centrality and betweenness centrality are implemented for graph analysis.^20,22,23^

## Results and discussion

Data analysis is performed on the data obtained from cobalt catalyzed hydroformylation reaction calculations. The data set consists of 8558 data points with the following information in the columns: reactant node number, product node number, equilibrium energy of the reactant, equilibrium energy of the product, and activation energy barrier. Node number and activation energy barrier are treated as nodes and edges in the network, respectively. The data is treated as a directed graph where reversing the node results in different activation energy barriers. Note that the data and corresponding structural information are listed in the ESI.† Frequency analysis reveals that node number 54 appears 55 times within the 8558 data points. In particular, node number 54, which represents cobalt tetracarbonyl hydride HCo(CO)_4_ with H_2_ and C_2_H_4_ molecules, is found to be the node with the highest frequency within the map. Understanding which nodes have high frequency in the reaction network allows one to better understand the initial step taken within the reaction as high frequency indicates that many nodes visit this node. Given its frequency, one can therefore see that the molecules represented in node number 54 experience a high level of traffic within the network and thus can be seen as a key step of hydroformylation.

**Fig. 1:**
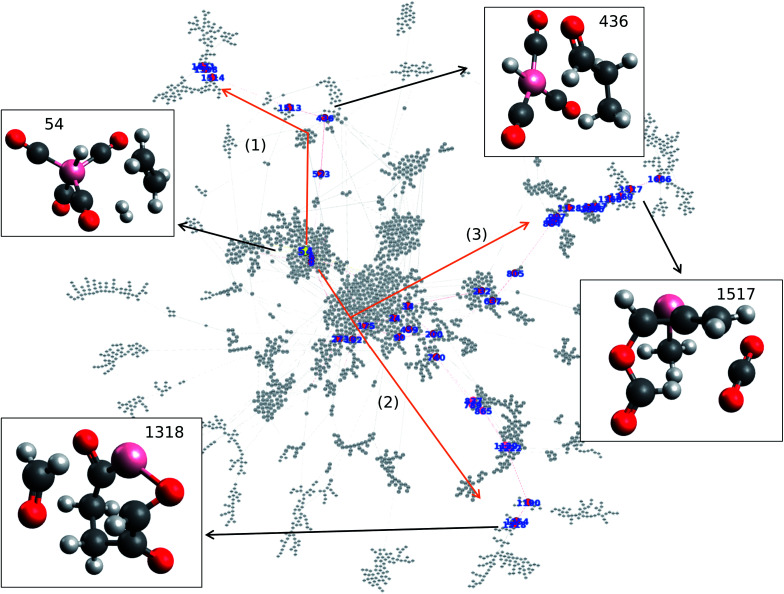
Visualization of the hydroformylation reaction network *via* Force Atlas 2. Structures of reactant and key products are also represented. Color code: pink: cobalt; gray: carbon; white: hydrogen; red: oxygen.

Network visualization is performed in order to represent the calculated hydroformylation reaction as a network. In particular, the Force Atlas 2 algorithm is used in order to visualize the network as shown in Fig. 1.^20^ Network visualization is informed by the continuous algorithm and is force-directed where nodes repel each other while edges attract their respective nodes, making node placement dependent on the other nodes present within the network. Fig. 1 shows the overall reaction network of hydroformylation created by the AFIR method where the reaction network traces how the AFIR method navigates the reaction. Fig. 1 demonstrates that some nodes form clusters at the center of the network while other groups form a branch-like structure that stems out from the center of the network. Note that node numbers 26, 145, 1856, and 1812 are isolated from the network as a result of SCF convergence failure that occurred on paths to these nodes.

Here, the question arises concerning how the reactant, product, and reaction paths connecting them will be identified within the reaction network as shown in Fig. 1. In order to find the key nodes within the reaction network, network analysis is performed in terms of graph theory. In particular, harmonic closeness centrality and betweenness centrality are explored where closeness and betweenness represent how close a node is to other nodes and which nodes control the network, respectively.^22^ In other words, one can consider that harmonic closeness centrality can help indicate energetically stable structures while betweenness centrality can help indicate key intermediate compounds in the reaction. Harmonic closeness demonstrates that node number 54 has the highest score, indicating that node 54 accesses many neighboring nodes and has good agreement with the observation that node 54 has the highest frequency within the network. Note that node 54 is colored in yellow in Fig. 1. HCo(CO)_4_, the compound represented by node 54, has been previously reported to be an active catalyst for hydroformylation where the dissociation of CO from HCo(CO)_4_ is considered as the initial step for hydroformylation.^24^ Hence, analyzing closeness centrality helps determine the reactant within the calculated hydroformylation reaction network.

Similarly, betweenness centrality is investigated where the top 40 betweenness centrality nodes are selected and colored in red as shown in Fig. 1. Please see the ESI† for the top 40 betweenness centrality nodes. It is surprising to find that three paths appear when connecting the top 40 betweenness centrality nodes as shown in Fig. 1. More importantly, each path contains key molecules where paths (1), (2), and (3) result in aldehydes, formaldehyde (CH_2_O), and carbon dioxide (CO_2_), respectively. Given that hydroformylation is a reaction that produces aldehydes, this result therefore shows that betweenness centrality can be used to help identify reaction paths for aldehyde formation from node 54 (which contains HCo(CO)_4_) without kinetics.

**Fig. 2:**
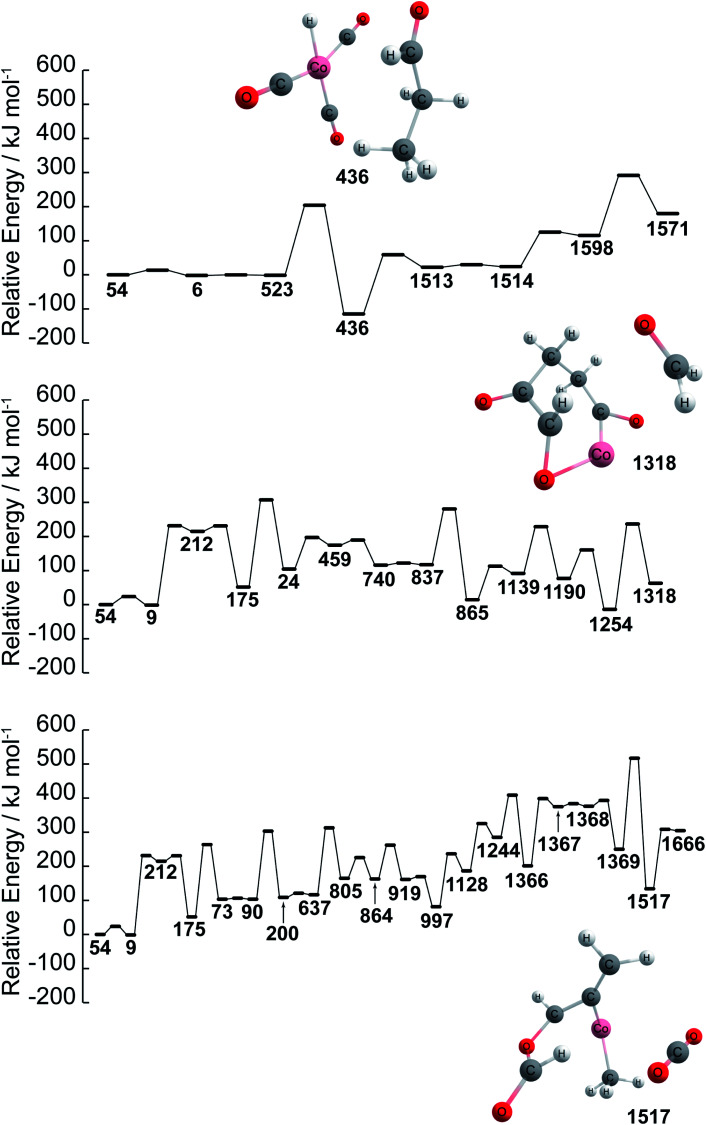
Energy profiles of paths (1), (2), and (3) found by betweenness centrality. *E*_a_ is the activation energy barrier. Structures of reactant and key products are also represented. Color code: pink: cobalt; gray: carbon; white: hydrogen; red: oxygen. Note that the energies path is searched and calculated by AFIR with Gaussian 16 and numbers represent the node number in Fig. 1.

Further details of paths (1), (2), and (3) in Fig. 1 are investigated in terms of the energy profiles as shown in Fig. 2. The energy profile of path (1) indicates that aldehydes (node 436) are formed in the node order 54 → 6 → 523 → 436 where an activation barrier of 204.05 kJ mol^−1^ is required when attempting to move from nodes 523 to 436. In the same fashion, paths (2) and (3) are analyzed *via* an energy profile. However, these paths encounter multiple high activation energy barriers and endothermic reactions when attempting to arrive at CH_2_O (node 1254) and CO_2_ (node 1517) in path (2) and path (3), respectively. Note that node 212 is not in top 40 ranking nodes with high betweenness centrality. Therefore, paths (2) and (3) (illustrated in Fig. 1) can be considered to be unlikely to occur while path (1) is energetically favored. Although the actual reaction path could be more complex with kinetic analysis, data science has provided a near-instant method of providing potential candidates for reactants, products, and reaction paths encountered within a complex reaction network. This approach therefore accelerates the identification of reaction paths within a reaction network without additional kinetics analysis. It must be noted that the frequency of nodes as well as betweenness centrality analysis are able to detect key nodes based on the structure of the network shown in Fig. 1 created by a development version of the GRRM program (version on April 9th, 2020), therefore, different network analysis might be required depending on the structure of network. In other words, the proposed approach can act as the first step towards deeper kinetic analysis into a reaction network and provide insight into where further investigation can occur.

**Fig. 3:**
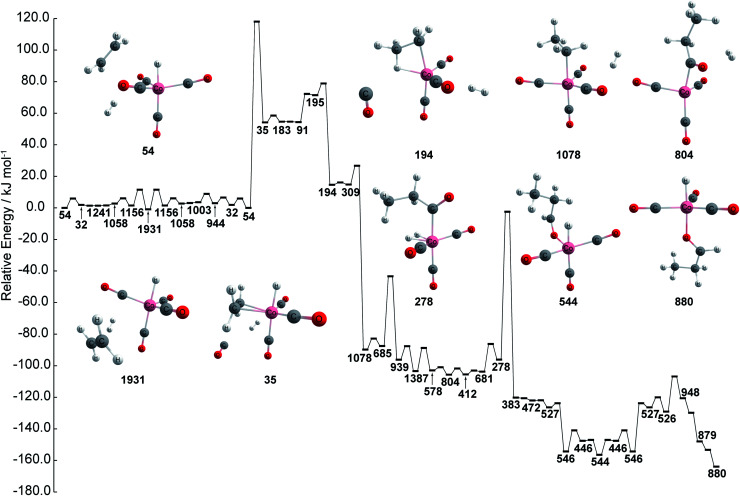
Energy profile of stable path by kinetic analysis. Color code: pink: cobalt; gray: carbon; white: hydrogen; red: oxygen. Note that the energies path is searched and calculated by AFIR with Gaussian 16 and numbers represent the node number in Fig. 1.

The chemical reaction network is then kinetically investigated in order to compare kinetics against the proposed reaction path created using graph theory. The kinetically most feasible path from node 54 to the most stable catalyst–product complex 880 is extracted from the network and depicted in Fig. 3. The path is obtained by combining the shortest path in terms of overall rate constants with local equilibration paths. The local equilibration paths can be seen in the processes from 54 to 1931 and from 527 to 544. The path consists of many steps that include bond reorganization steps and fast steps such as pseudo rotation and conformation change. As can be seen in Fig. 3, the energy profile has three regions: (a) an initial region including node 54 which represents HCo(CO)_4_ + C_2_H_4_ + H_2_, (b) the intermediate region including node 194 which represents CH_3_CH_2_Co(CO)_3_ + CO + H_2_, including node 1078 which represents CH_3_CH_2_Co(CO)_4_ + H_2_, CH_3_CH_2_C(O)Co(CO)_3_ + H_2_, and (c) the final region which includes node 446 which represents HCo(CO)_3_ + CH_3_CH_2_CHO. This path shows agreement with the well-known Heck–Breslow mechanism, which justifies the use of this network in this study.^14^ Additionally, aldehyde is found in both the graph network and the kinetic study, where aldehyde is found to be produced at node 436 within the network while aldehyde is found to be produced at node 880 during the kinetic investigation. Thus, these results show that the kinetic study and chemical reaction network are both capable of finding nodes where aldehyde is produced.

Here, one can also understand that the closeness centrality and betweenness centrality of the network shown in Fig. 1 reflects the search procedure used in the SC-AFIR algorithm. First, when *Λ*_i_ is used as an index to rank local minimum structures, the preference in a group that reaches equilibrium in a shorter timescale than *t*_MAX_ is dependent on the Boltzmann distribution at *T*_R_. At the start of the search, only complexes among HCo(CO)_3_ + CO + C_2_H_4_ + H_2_ are considered and paths are computed from these structures. Once the structures in the intermediate region are found, searches are done preferentially from structures in the intermediate region since the structures in the initial region can transition to the intermediate region within *t*_MAX_. Finally, searches are done preferentially from structures in the final region since the structures in the initial and intermediate regions can transition to the final region within *t*_MAX_. It should be noted that many other possibilities that originate from the initial region are searched as the other regions are unknown at the start. This can account for why structures having the highest closeness centrality are found within the initial region. Node 54 is quasi-symmetric, having C=C and H–H almost on the plane of H, Co, and C in the axial CO, making it a possible transit point among other HCo(CO)_4_ + C_2_H_4_ + H_2_ complexes in the initial region. Therefore, the closeness centrality is useful for identifying the most important structure within the initial region. As noted, the SC-AFIR searched various possibilities originating from the initial region and created many local areas within the network. By definition, a node that has high betweenness centrality is a node that can be viewed as having more control in the network as many paths lead through it. Given this, structures that have high betweenness centrality should correspond to key intermediates within paths that connect to different areas of the network. Various different chemical transformations are able to be identified by tracing nodes having high betweenness centrality. Identifying possible intermediates, regardless of their kinetic importance, is very important for actual mechanism studies on chemical reactions. Hence, betweenness centrality can be powerful for identifying key intermediates. Chemical reaction network has become possible to create by combining the first principles calculation with data science. However, it has been a challenge to extract knowledge from the network due to the high complexity of the chemical reaction. Here, data science, graph theory in particular, is found to be a powerful approach for quickly extracting knowledge from the reaction network without any kinetics analysis. Thus, combining graph theory and the first principles calculations helps accelerate the determination of the reaction path in a chemical reaction network.

## Conclusion

In summary, data science is implemented to determine the reaction path in a calculated reaction network. In particular, cobalt catalyzed hydroformylation is selected as a prototype reaction where artificial force induced reaction within the first principles calculations is implemented to create a hydroformylation reaction network. Data science, graph theory, unveils the frequency of nodes as well as closeness centrality determining the reactant which is found to be cobalt tetracarbonyl hydride HCo(CO)_4_. Furthermore, betweenness centrality reveals the 3 reaction paths which lead to the formation of aldehyde, CH_2_O, and CO_2_ where the energy profile indicates that the formation of aldehyde is energetically favored. Thus, it is proposed that data science accelerates identification of the reactant, product, and reaction path from the complex reaction network without kinetics. This approach would act as a first step for understanding a complex reaction network towards further kinetic understanding of a chemical reaction.

## Author contributions

K. T. performed network and data analysis. S. M. calculated the reaction network.

## Conflicts of interest

There are no conflicts to declare.

## Supplementary Material

RA-011-D1RA03395F-s001

RA-011-D1RA03395F-s002

RA-011-D1RA03395F-s003

RA-011-D1RA03395F-s004

RA-011-D1RA03395F-s005

RA-011-D1RA03395F-s006

RA-011-D1RA03395F-s007

RA-011-D1RA03395F-s008

RA-011-D1RA03395F-s009

RA-011-D1RA03395F-s010

RA-011-D1RA03395F-s011

RA-011-D1RA03395F-s012

RA-011-D1RA03395F-s013

RA-011-D1RA03395F-s014

RA-011-D1RA03395F-s015

RA-011-D1RA03395F-s016

RA-011-D1RA03395F-s017

RA-011-D1RA03395F-s018

RA-011-D1RA03395F-s019

RA-011-D1RA03395F-s020

RA-011-D1RA03395F-s021

RA-011-D1RA03395F-s022

RA-011-D1RA03395F-s023

RA-011-D1RA03395F-s024

RA-011-D1RA03395F-s025

RA-011-D1RA03395F-s026

RA-011-D1RA03395F-s027

RA-011-D1RA03395F-s028

RA-011-D1RA03395F-s029

RA-011-D1RA03395F-s030

RA-011-D1RA03395F-s031

RA-011-D1RA03395F-s032

RA-011-D1RA03395F-s033

RA-011-D1RA03395F-s034

RA-011-D1RA03395F-s035

RA-011-D1RA03395F-s036

RA-011-D1RA03395F-s037

RA-011-D1RA03395F-s038

RA-011-D1RA03395F-s039

RA-011-D1RA03395F-s040

RA-011-D1RA03395F-s041

RA-011-D1RA03395F-s042

RA-011-D1RA03395F-s043

RA-011-D1RA03395F-s044

RA-011-D1RA03395F-s045

RA-011-D1RA03395F-s046

RA-011-D1RA03395F-s047

RA-011-D1RA03395F-s048

RA-011-D1RA03395F-s049

RA-011-D1RA03395F-s050

RA-011-D1RA03395F-s051

RA-011-D1RA03395F-s052

RA-011-D1RA03395F-s053

RA-011-D1RA03395F-s054

RA-011-D1RA03395F-s055

RA-011-D1RA03395F-s056

RA-011-D1RA03395F-s057

RA-011-D1RA03395F-s058

RA-011-D1RA03395F-s059

RA-011-D1RA03395F-s060

RA-011-D1RA03395F-s061

RA-011-D1RA03395F-s062

RA-011-D1RA03395F-s063

RA-011-D1RA03395F-s064

RA-011-D1RA03395F-s065

RA-011-D1RA03395F-s066

RA-011-D1RA03395F-s067

RA-011-D1RA03395F-s068

RA-011-D1RA03395F-s069

RA-011-D1RA03395F-s070

RA-011-D1RA03395F-s071

RA-011-D1RA03395F-s072

RA-011-D1RA03395F-s073

RA-011-D1RA03395F-s074

RA-011-D1RA03395F-s075

RA-011-D1RA03395F-s076

RA-011-D1RA03395F-s077

RA-011-D1RA03395F-s078

RA-011-D1RA03395F-s079

RA-011-D1RA03395F-s080

RA-011-D1RA03395F-s081

RA-011-D1RA03395F-s082

RA-011-D1RA03395F-s083

RA-011-D1RA03395F-s084

RA-011-D1RA03395F-s085

RA-011-D1RA03395F-s086

RA-011-D1RA03395F-s087

RA-011-D1RA03395F-s088

RA-011-D1RA03395F-s089

RA-011-D1RA03395F-s090

RA-011-D1RA03395F-s091

RA-011-D1RA03395F-s092

RA-011-D1RA03395F-s093

RA-011-D1RA03395F-s094

RA-011-D1RA03395F-s095

RA-011-D1RA03395F-s096

RA-011-D1RA03395F-s097

RA-011-D1RA03395F-s098

RA-011-D1RA03395F-s099

RA-011-D1RA03395F-s100

RA-011-D1RA03395F-s101

RA-011-D1RA03395F-s102

RA-011-D1RA03395F-s103

RA-011-D1RA03395F-s104

RA-011-D1RA03395F-s105

RA-011-D1RA03395F-s106

RA-011-D1RA03395F-s107

RA-011-D1RA03395F-s108

RA-011-D1RA03395F-s109

RA-011-D1RA03395F-s110

RA-011-D1RA03395F-s111

RA-011-D1RA03395F-s112

RA-011-D1RA03395F-s113

RA-011-D1RA03395F-s114

RA-011-D1RA03395F-s115

RA-011-D1RA03395F-s116

RA-011-D1RA03395F-s117

RA-011-D1RA03395F-s118

RA-011-D1RA03395F-s119

RA-011-D1RA03395F-s120

RA-011-D1RA03395F-s121

RA-011-D1RA03395F-s122

RA-011-D1RA03395F-s123

RA-011-D1RA03395F-s124

RA-011-D1RA03395F-s125

RA-011-D1RA03395F-s126

RA-011-D1RA03395F-s127

RA-011-D1RA03395F-s128

RA-011-D1RA03395F-s129

RA-011-D1RA03395F-s130

RA-011-D1RA03395F-s131

RA-011-D1RA03395F-s132

RA-011-D1RA03395F-s133

RA-011-D1RA03395F-s134

RA-011-D1RA03395F-s135

RA-011-D1RA03395F-s136

RA-011-D1RA03395F-s137

RA-011-D1RA03395F-s138

RA-011-D1RA03395F-s139

RA-011-D1RA03395F-s140

RA-011-D1RA03395F-s141

RA-011-D1RA03395F-s142

RA-011-D1RA03395F-s143

RA-011-D1RA03395F-s144

RA-011-D1RA03395F-s145

RA-011-D1RA03395F-s146

RA-011-D1RA03395F-s147

RA-011-D1RA03395F-s148

RA-011-D1RA03395F-s149

RA-011-D1RA03395F-s150

RA-011-D1RA03395F-s151

RA-011-D1RA03395F-s152

RA-011-D1RA03395F-s153

RA-011-D1RA03395F-s154

RA-011-D1RA03395F-s155

RA-011-D1RA03395F-s156

RA-011-D1RA03395F-s157

RA-011-D1RA03395F-s158

RA-011-D1RA03395F-s159

RA-011-D1RA03395F-s160

RA-011-D1RA03395F-s161

RA-011-D1RA03395F-s162

RA-011-D1RA03395F-s163

RA-011-D1RA03395F-s164

RA-011-D1RA03395F-s165

RA-011-D1RA03395F-s166

RA-011-D1RA03395F-s167

RA-011-D1RA03395F-s168

RA-011-D1RA03395F-s169

RA-011-D1RA03395F-s170

RA-011-D1RA03395F-s171

RA-011-D1RA03395F-s172

RA-011-D1RA03395F-s173

RA-011-D1RA03395F-s174

RA-011-D1RA03395F-s175

RA-011-D1RA03395F-s176

RA-011-D1RA03395F-s177

RA-011-D1RA03395F-s178

RA-011-D1RA03395F-s179

RA-011-D1RA03395F-s180

RA-011-D1RA03395F-s181

RA-011-D1RA03395F-s182

RA-011-D1RA03395F-s183

RA-011-D1RA03395F-s184

RA-011-D1RA03395F-s185

RA-011-D1RA03395F-s186

RA-011-D1RA03395F-s187

RA-011-D1RA03395F-s188

RA-011-D1RA03395F-s189

RA-011-D1RA03395F-s190

RA-011-D1RA03395F-s191

RA-011-D1RA03395F-s192

RA-011-D1RA03395F-s193

RA-011-D1RA03395F-s194

RA-011-D1RA03395F-s195

RA-011-D1RA03395F-s196

RA-011-D1RA03395F-s197

RA-011-D1RA03395F-s198

RA-011-D1RA03395F-s199

RA-011-D1RA03395F-s200

RA-011-D1RA03395F-s201

RA-011-D1RA03395F-s202

RA-011-D1RA03395F-s203

RA-011-D1RA03395F-s204

RA-011-D1RA03395F-s205

RA-011-D1RA03395F-s206

RA-011-D1RA03395F-s207

RA-011-D1RA03395F-s208

RA-011-D1RA03395F-s209

RA-011-D1RA03395F-s210

RA-011-D1RA03395F-s211

RA-011-D1RA03395F-s212

RA-011-D1RA03395F-s213

RA-011-D1RA03395F-s214

RA-011-D1RA03395F-s215

RA-011-D1RA03395F-s216

RA-011-D1RA03395F-s217

RA-011-D1RA03395F-s218

RA-011-D1RA03395F-s219

RA-011-D1RA03395F-s220

RA-011-D1RA03395F-s221

RA-011-D1RA03395F-s222

RA-011-D1RA03395F-s223

RA-011-D1RA03395F-s224

RA-011-D1RA03395F-s225

RA-011-D1RA03395F-s226

RA-011-D1RA03395F-s227

RA-011-D1RA03395F-s228

RA-011-D1RA03395F-s229

RA-011-D1RA03395F-s230

RA-011-D1RA03395F-s231

RA-011-D1RA03395F-s232

RA-011-D1RA03395F-s233

RA-011-D1RA03395F-s234

RA-011-D1RA03395F-s235

RA-011-D1RA03395F-s236

RA-011-D1RA03395F-s237

RA-011-D1RA03395F-s238

RA-011-D1RA03395F-s239

RA-011-D1RA03395F-s240

RA-011-D1RA03395F-s241

RA-011-D1RA03395F-s242

RA-011-D1RA03395F-s243

RA-011-D1RA03395F-s244

RA-011-D1RA03395F-s245

RA-011-D1RA03395F-s246

RA-011-D1RA03395F-s247

RA-011-D1RA03395F-s248

RA-011-D1RA03395F-s249

RA-011-D1RA03395F-s250

RA-011-D1RA03395F-s251

RA-011-D1RA03395F-s252

RA-011-D1RA03395F-s253

RA-011-D1RA03395F-s254

RA-011-D1RA03395F-s255

RA-011-D1RA03395F-s256

RA-011-D1RA03395F-s257

RA-011-D1RA03395F-s258

RA-011-D1RA03395F-s259

RA-011-D1RA03395F-s260

RA-011-D1RA03395F-s261

RA-011-D1RA03395F-s262

RA-011-D1RA03395F-s263

RA-011-D1RA03395F-s264

RA-011-D1RA03395F-s265

RA-011-D1RA03395F-s266

RA-011-D1RA03395F-s267

RA-011-D1RA03395F-s268

RA-011-D1RA03395F-s269

RA-011-D1RA03395F-s270

RA-011-D1RA03395F-s271

RA-011-D1RA03395F-s272

RA-011-D1RA03395F-s273

RA-011-D1RA03395F-s274

RA-011-D1RA03395F-s275

RA-011-D1RA03395F-s276

RA-011-D1RA03395F-s277

RA-011-D1RA03395F-s278

RA-011-D1RA03395F-s279

RA-011-D1RA03395F-s280

RA-011-D1RA03395F-s281

RA-011-D1RA03395F-s282

RA-011-D1RA03395F-s283

RA-011-D1RA03395F-s284

RA-011-D1RA03395F-s285

RA-011-D1RA03395F-s286

RA-011-D1RA03395F-s287

RA-011-D1RA03395F-s288

RA-011-D1RA03395F-s289

RA-011-D1RA03395F-s290

RA-011-D1RA03395F-s291

RA-011-D1RA03395F-s292

RA-011-D1RA03395F-s293

RA-011-D1RA03395F-s294

RA-011-D1RA03395F-s295

RA-011-D1RA03395F-s296

RA-011-D1RA03395F-s297

RA-011-D1RA03395F-s298

RA-011-D1RA03395F-s299

RA-011-D1RA03395F-s300

RA-011-D1RA03395F-s301

RA-011-D1RA03395F-s302

RA-011-D1RA03395F-s303

RA-011-D1RA03395F-s304

RA-011-D1RA03395F-s305

RA-011-D1RA03395F-s306

RA-011-D1RA03395F-s307

RA-011-D1RA03395F-s308

RA-011-D1RA03395F-s309

RA-011-D1RA03395F-s310

RA-011-D1RA03395F-s311

RA-011-D1RA03395F-s312

RA-011-D1RA03395F-s313

RA-011-D1RA03395F-s314

RA-011-D1RA03395F-s315

RA-011-D1RA03395F-s316

RA-011-D1RA03395F-s317

RA-011-D1RA03395F-s318

RA-011-D1RA03395F-s319

RA-011-D1RA03395F-s320

RA-011-D1RA03395F-s321

RA-011-D1RA03395F-s322

RA-011-D1RA03395F-s323

RA-011-D1RA03395F-s324

RA-011-D1RA03395F-s325

RA-011-D1RA03395F-s326

RA-011-D1RA03395F-s327

RA-011-D1RA03395F-s328

RA-011-D1RA03395F-s329

RA-011-D1RA03395F-s330

RA-011-D1RA03395F-s331

RA-011-D1RA03395F-s332

RA-011-D1RA03395F-s333

RA-011-D1RA03395F-s334

RA-011-D1RA03395F-s335

RA-011-D1RA03395F-s336

RA-011-D1RA03395F-s337

RA-011-D1RA03395F-s338

RA-011-D1RA03395F-s339

RA-011-D1RA03395F-s340

RA-011-D1RA03395F-s341

RA-011-D1RA03395F-s342

RA-011-D1RA03395F-s343

RA-011-D1RA03395F-s344

RA-011-D1RA03395F-s345

RA-011-D1RA03395F-s346

RA-011-D1RA03395F-s347

RA-011-D1RA03395F-s348

RA-011-D1RA03395F-s349

RA-011-D1RA03395F-s350

RA-011-D1RA03395F-s351

RA-011-D1RA03395F-s352

RA-011-D1RA03395F-s353

RA-011-D1RA03395F-s354

RA-011-D1RA03395F-s355

RA-011-D1RA03395F-s356

RA-011-D1RA03395F-s357

RA-011-D1RA03395F-s358

RA-011-D1RA03395F-s359

RA-011-D1RA03395F-s360

RA-011-D1RA03395F-s361

RA-011-D1RA03395F-s362

RA-011-D1RA03395F-s363

RA-011-D1RA03395F-s364

RA-011-D1RA03395F-s365

RA-011-D1RA03395F-s366

RA-011-D1RA03395F-s367

RA-011-D1RA03395F-s368

RA-011-D1RA03395F-s369

RA-011-D1RA03395F-s370

RA-011-D1RA03395F-s371

RA-011-D1RA03395F-s372

RA-011-D1RA03395F-s373

RA-011-D1RA03395F-s374

RA-011-D1RA03395F-s375

RA-011-D1RA03395F-s376

RA-011-D1RA03395F-s377

RA-011-D1RA03395F-s378

RA-011-D1RA03395F-s379

RA-011-D1RA03395F-s380

RA-011-D1RA03395F-s381

RA-011-D1RA03395F-s382

RA-011-D1RA03395F-s383

RA-011-D1RA03395F-s384

RA-011-D1RA03395F-s385

RA-011-D1RA03395F-s386

RA-011-D1RA03395F-s387

RA-011-D1RA03395F-s388

RA-011-D1RA03395F-s389

RA-011-D1RA03395F-s390

RA-011-D1RA03395F-s391

RA-011-D1RA03395F-s392

RA-011-D1RA03395F-s393

RA-011-D1RA03395F-s394

RA-011-D1RA03395F-s395

RA-011-D1RA03395F-s396

RA-011-D1RA03395F-s397

RA-011-D1RA03395F-s398

RA-011-D1RA03395F-s399

RA-011-D1RA03395F-s400

RA-011-D1RA03395F-s401

RA-011-D1RA03395F-s402

RA-011-D1RA03395F-s403

RA-011-D1RA03395F-s404

RA-011-D1RA03395F-s405

RA-011-D1RA03395F-s406

RA-011-D1RA03395F-s407

RA-011-D1RA03395F-s408

RA-011-D1RA03395F-s409

RA-011-D1RA03395F-s410

RA-011-D1RA03395F-s411

RA-011-D1RA03395F-s412

RA-011-D1RA03395F-s413

RA-011-D1RA03395F-s414

RA-011-D1RA03395F-s415

RA-011-D1RA03395F-s416

RA-011-D1RA03395F-s417

RA-011-D1RA03395F-s418

RA-011-D1RA03395F-s419

RA-011-D1RA03395F-s420

RA-011-D1RA03395F-s421

RA-011-D1RA03395F-s422

RA-011-D1RA03395F-s423

RA-011-D1RA03395F-s424

RA-011-D1RA03395F-s425

RA-011-D1RA03395F-s426

RA-011-D1RA03395F-s427

RA-011-D1RA03395F-s428

RA-011-D1RA03395F-s429

RA-011-D1RA03395F-s430

RA-011-D1RA03395F-s431

RA-011-D1RA03395F-s432

RA-011-D1RA03395F-s433

RA-011-D1RA03395F-s434

RA-011-D1RA03395F-s435

RA-011-D1RA03395F-s436

RA-011-D1RA03395F-s437

RA-011-D1RA03395F-s438

RA-011-D1RA03395F-s439

RA-011-D1RA03395F-s440

RA-011-D1RA03395F-s441

RA-011-D1RA03395F-s442

RA-011-D1RA03395F-s443

RA-011-D1RA03395F-s444

RA-011-D1RA03395F-s445

RA-011-D1RA03395F-s446

RA-011-D1RA03395F-s447

RA-011-D1RA03395F-s448

RA-011-D1RA03395F-s449

RA-011-D1RA03395F-s450

RA-011-D1RA03395F-s451

RA-011-D1RA03395F-s452

RA-011-D1RA03395F-s453

RA-011-D1RA03395F-s454

RA-011-D1RA03395F-s455

RA-011-D1RA03395F-s456

RA-011-D1RA03395F-s457

RA-011-D1RA03395F-s458

RA-011-D1RA03395F-s459

RA-011-D1RA03395F-s460

RA-011-D1RA03395F-s461

RA-011-D1RA03395F-s462

RA-011-D1RA03395F-s463

RA-011-D1RA03395F-s464

RA-011-D1RA03395F-s465

RA-011-D1RA03395F-s466

RA-011-D1RA03395F-s467

RA-011-D1RA03395F-s468

RA-011-D1RA03395F-s469

RA-011-D1RA03395F-s470

RA-011-D1RA03395F-s471

RA-011-D1RA03395F-s472

RA-011-D1RA03395F-s473

RA-011-D1RA03395F-s474

RA-011-D1RA03395F-s475

RA-011-D1RA03395F-s476

RA-011-D1RA03395F-s477

RA-011-D1RA03395F-s478

RA-011-D1RA03395F-s479

RA-011-D1RA03395F-s480

RA-011-D1RA03395F-s481

RA-011-D1RA03395F-s482

RA-011-D1RA03395F-s483

RA-011-D1RA03395F-s484

RA-011-D1RA03395F-s485

RA-011-D1RA03395F-s486

RA-011-D1RA03395F-s487

RA-011-D1RA03395F-s488

RA-011-D1RA03395F-s489

RA-011-D1RA03395F-s490

RA-011-D1RA03395F-s491

RA-011-D1RA03395F-s492

RA-011-D1RA03395F-s493

RA-011-D1RA03395F-s494

RA-011-D1RA03395F-s495

RA-011-D1RA03395F-s496

RA-011-D1RA03395F-s497

RA-011-D1RA03395F-s498

RA-011-D1RA03395F-s499

RA-011-D1RA03395F-s500

RA-011-D1RA03395F-s501

RA-011-D1RA03395F-s502

RA-011-D1RA03395F-s503

RA-011-D1RA03395F-s504

RA-011-D1RA03395F-s505

RA-011-D1RA03395F-s506

RA-011-D1RA03395F-s507

RA-011-D1RA03395F-s508

RA-011-D1RA03395F-s509

RA-011-D1RA03395F-s510

RA-011-D1RA03395F-s511

RA-011-D1RA03395F-s512

RA-011-D1RA03395F-s513

RA-011-D1RA03395F-s514

RA-011-D1RA03395F-s515

RA-011-D1RA03395F-s516

RA-011-D1RA03395F-s517

RA-011-D1RA03395F-s518

RA-011-D1RA03395F-s519

RA-011-D1RA03395F-s520

RA-011-D1RA03395F-s521

RA-011-D1RA03395F-s522

RA-011-D1RA03395F-s523

RA-011-D1RA03395F-s524

RA-011-D1RA03395F-s525

RA-011-D1RA03395F-s526

RA-011-D1RA03395F-s527

RA-011-D1RA03395F-s528

RA-011-D1RA03395F-s529

RA-011-D1RA03395F-s530

RA-011-D1RA03395F-s531

RA-011-D1RA03395F-s532

RA-011-D1RA03395F-s533

RA-011-D1RA03395F-s534

RA-011-D1RA03395F-s535

RA-011-D1RA03395F-s536

RA-011-D1RA03395F-s537

RA-011-D1RA03395F-s538

RA-011-D1RA03395F-s539

RA-011-D1RA03395F-s540

RA-011-D1RA03395F-s541

RA-011-D1RA03395F-s542

RA-011-D1RA03395F-s543

RA-011-D1RA03395F-s544

RA-011-D1RA03395F-s545

RA-011-D1RA03395F-s546

RA-011-D1RA03395F-s547

RA-011-D1RA03395F-s548

RA-011-D1RA03395F-s549

RA-011-D1RA03395F-s550

RA-011-D1RA03395F-s551

RA-011-D1RA03395F-s552

RA-011-D1RA03395F-s553

RA-011-D1RA03395F-s554

RA-011-D1RA03395F-s555

RA-011-D1RA03395F-s556

RA-011-D1RA03395F-s557

RA-011-D1RA03395F-s558

RA-011-D1RA03395F-s559

RA-011-D1RA03395F-s560

RA-011-D1RA03395F-s561

RA-011-D1RA03395F-s562

RA-011-D1RA03395F-s563

RA-011-D1RA03395F-s564

RA-011-D1RA03395F-s565

RA-011-D1RA03395F-s566

RA-011-D1RA03395F-s567

RA-011-D1RA03395F-s568

RA-011-D1RA03395F-s569

RA-011-D1RA03395F-s570

RA-011-D1RA03395F-s571

RA-011-D1RA03395F-s572

RA-011-D1RA03395F-s573

RA-011-D1RA03395F-s574

RA-011-D1RA03395F-s575

RA-011-D1RA03395F-s576

RA-011-D1RA03395F-s577

RA-011-D1RA03395F-s578

RA-011-D1RA03395F-s579

RA-011-D1RA03395F-s580

RA-011-D1RA03395F-s581

RA-011-D1RA03395F-s582

RA-011-D1RA03395F-s583

RA-011-D1RA03395F-s584

RA-011-D1RA03395F-s585

RA-011-D1RA03395F-s586

RA-011-D1RA03395F-s587

RA-011-D1RA03395F-s588

RA-011-D1RA03395F-s589

RA-011-D1RA03395F-s590

RA-011-D1RA03395F-s591

RA-011-D1RA03395F-s592

RA-011-D1RA03395F-s593

RA-011-D1RA03395F-s594

RA-011-D1RA03395F-s595

RA-011-D1RA03395F-s596

RA-011-D1RA03395F-s597

RA-011-D1RA03395F-s598

RA-011-D1RA03395F-s599

RA-011-D1RA03395F-s600

RA-011-D1RA03395F-s601

RA-011-D1RA03395F-s602

RA-011-D1RA03395F-s603

RA-011-D1RA03395F-s604

RA-011-D1RA03395F-s605

RA-011-D1RA03395F-s606

RA-011-D1RA03395F-s607

RA-011-D1RA03395F-s608

RA-011-D1RA03395F-s609

RA-011-D1RA03395F-s610

RA-011-D1RA03395F-s611

RA-011-D1RA03395F-s612

RA-011-D1RA03395F-s613

RA-011-D1RA03395F-s614

RA-011-D1RA03395F-s615

RA-011-D1RA03395F-s616

RA-011-D1RA03395F-s617

RA-011-D1RA03395F-s618

RA-011-D1RA03395F-s619

RA-011-D1RA03395F-s620

RA-011-D1RA03395F-s621

RA-011-D1RA03395F-s622

RA-011-D1RA03395F-s623

RA-011-D1RA03395F-s624

RA-011-D1RA03395F-s625

RA-011-D1RA03395F-s626

RA-011-D1RA03395F-s627

RA-011-D1RA03395F-s628

RA-011-D1RA03395F-s629

RA-011-D1RA03395F-s630

RA-011-D1RA03395F-s631

RA-011-D1RA03395F-s632

RA-011-D1RA03395F-s633

RA-011-D1RA03395F-s634

RA-011-D1RA03395F-s635

RA-011-D1RA03395F-s636

RA-011-D1RA03395F-s637

RA-011-D1RA03395F-s638

RA-011-D1RA03395F-s639

RA-011-D1RA03395F-s640

RA-011-D1RA03395F-s641

RA-011-D1RA03395F-s642

RA-011-D1RA03395F-s643

RA-011-D1RA03395F-s644

RA-011-D1RA03395F-s645

RA-011-D1RA03395F-s646

RA-011-D1RA03395F-s647

RA-011-D1RA03395F-s648

RA-011-D1RA03395F-s649

RA-011-D1RA03395F-s650

RA-011-D1RA03395F-s651

RA-011-D1RA03395F-s652

RA-011-D1RA03395F-s653

RA-011-D1RA03395F-s654

RA-011-D1RA03395F-s655

RA-011-D1RA03395F-s656

RA-011-D1RA03395F-s657

RA-011-D1RA03395F-s658

RA-011-D1RA03395F-s659

RA-011-D1RA03395F-s660

RA-011-D1RA03395F-s661

RA-011-D1RA03395F-s662

RA-011-D1RA03395F-s663

RA-011-D1RA03395F-s664

RA-011-D1RA03395F-s665

RA-011-D1RA03395F-s666

RA-011-D1RA03395F-s667

RA-011-D1RA03395F-s668

RA-011-D1RA03395F-s669

RA-011-D1RA03395F-s670

RA-011-D1RA03395F-s671

RA-011-D1RA03395F-s672

RA-011-D1RA03395F-s673

RA-011-D1RA03395F-s674

RA-011-D1RA03395F-s675

RA-011-D1RA03395F-s676

RA-011-D1RA03395F-s677

RA-011-D1RA03395F-s678

RA-011-D1RA03395F-s679

RA-011-D1RA03395F-s680

RA-011-D1RA03395F-s681

RA-011-D1RA03395F-s682

RA-011-D1RA03395F-s683

RA-011-D1RA03395F-s684

RA-011-D1RA03395F-s685

RA-011-D1RA03395F-s686

RA-011-D1RA03395F-s687

RA-011-D1RA03395F-s688

RA-011-D1RA03395F-s689

RA-011-D1RA03395F-s690

RA-011-D1RA03395F-s691

RA-011-D1RA03395F-s692

RA-011-D1RA03395F-s693

RA-011-D1RA03395F-s694

RA-011-D1RA03395F-s695

RA-011-D1RA03395F-s696

RA-011-D1RA03395F-s697

RA-011-D1RA03395F-s698

RA-011-D1RA03395F-s699

RA-011-D1RA03395F-s700

RA-011-D1RA03395F-s701

RA-011-D1RA03395F-s702

RA-011-D1RA03395F-s703

RA-011-D1RA03395F-s704

RA-011-D1RA03395F-s705

RA-011-D1RA03395F-s706

RA-011-D1RA03395F-s707

RA-011-D1RA03395F-s708

RA-011-D1RA03395F-s709

RA-011-D1RA03395F-s710

RA-011-D1RA03395F-s711

RA-011-D1RA03395F-s712

RA-011-D1RA03395F-s713

RA-011-D1RA03395F-s714

RA-011-D1RA03395F-s715

RA-011-D1RA03395F-s716

RA-011-D1RA03395F-s717

RA-011-D1RA03395F-s718

RA-011-D1RA03395F-s719

RA-011-D1RA03395F-s720

RA-011-D1RA03395F-s721

RA-011-D1RA03395F-s722

RA-011-D1RA03395F-s723

RA-011-D1RA03395F-s724

RA-011-D1RA03395F-s725

RA-011-D1RA03395F-s726

RA-011-D1RA03395F-s727

RA-011-D1RA03395F-s728

RA-011-D1RA03395F-s729

RA-011-D1RA03395F-s730

RA-011-D1RA03395F-s731

RA-011-D1RA03395F-s732

RA-011-D1RA03395F-s733

RA-011-D1RA03395F-s734

RA-011-D1RA03395F-s735

RA-011-D1RA03395F-s736

RA-011-D1RA03395F-s737

RA-011-D1RA03395F-s738

RA-011-D1RA03395F-s739

RA-011-D1RA03395F-s740

RA-011-D1RA03395F-s741

RA-011-D1RA03395F-s742

RA-011-D1RA03395F-s743

RA-011-D1RA03395F-s744

RA-011-D1RA03395F-s745

RA-011-D1RA03395F-s746

RA-011-D1RA03395F-s747

RA-011-D1RA03395F-s748

RA-011-D1RA03395F-s749

RA-011-D1RA03395F-s750

RA-011-D1RA03395F-s751

RA-011-D1RA03395F-s752

RA-011-D1RA03395F-s753

RA-011-D1RA03395F-s754

RA-011-D1RA03395F-s755

RA-011-D1RA03395F-s756

RA-011-D1RA03395F-s757

RA-011-D1RA03395F-s758

RA-011-D1RA03395F-s759

RA-011-D1RA03395F-s760

RA-011-D1RA03395F-s761

RA-011-D1RA03395F-s762

RA-011-D1RA03395F-s763

RA-011-D1RA03395F-s764

RA-011-D1RA03395F-s765

RA-011-D1RA03395F-s766

RA-011-D1RA03395F-s767

RA-011-D1RA03395F-s768

RA-011-D1RA03395F-s769

RA-011-D1RA03395F-s770

RA-011-D1RA03395F-s771

RA-011-D1RA03395F-s772

RA-011-D1RA03395F-s773

RA-011-D1RA03395F-s774

RA-011-D1RA03395F-s775

RA-011-D1RA03395F-s776

RA-011-D1RA03395F-s777

RA-011-D1RA03395F-s778

RA-011-D1RA03395F-s779

RA-011-D1RA03395F-s780

RA-011-D1RA03395F-s781

RA-011-D1RA03395F-s782

RA-011-D1RA03395F-s783

RA-011-D1RA03395F-s784

RA-011-D1RA03395F-s785

RA-011-D1RA03395F-s786

RA-011-D1RA03395F-s787

RA-011-D1RA03395F-s788

RA-011-D1RA03395F-s789

RA-011-D1RA03395F-s790

RA-011-D1RA03395F-s791

RA-011-D1RA03395F-s792

RA-011-D1RA03395F-s793

RA-011-D1RA03395F-s794

RA-011-D1RA03395F-s795

RA-011-D1RA03395F-s796

RA-011-D1RA03395F-s797

RA-011-D1RA03395F-s798

RA-011-D1RA03395F-s799

RA-011-D1RA03395F-s800

RA-011-D1RA03395F-s801

RA-011-D1RA03395F-s802

RA-011-D1RA03395F-s803

RA-011-D1RA03395F-s804

RA-011-D1RA03395F-s805

RA-011-D1RA03395F-s806

RA-011-D1RA03395F-s807

RA-011-D1RA03395F-s808

RA-011-D1RA03395F-s809

RA-011-D1RA03395F-s810

RA-011-D1RA03395F-s811

RA-011-D1RA03395F-s812

RA-011-D1RA03395F-s813

RA-011-D1RA03395F-s814

RA-011-D1RA03395F-s815

RA-011-D1RA03395F-s816

RA-011-D1RA03395F-s817

RA-011-D1RA03395F-s818

RA-011-D1RA03395F-s819

RA-011-D1RA03395F-s820

RA-011-D1RA03395F-s821

RA-011-D1RA03395F-s822

RA-011-D1RA03395F-s823

RA-011-D1RA03395F-s824

RA-011-D1RA03395F-s825

RA-011-D1RA03395F-s826

RA-011-D1RA03395F-s827

RA-011-D1RA03395F-s828

RA-011-D1RA03395F-s829

RA-011-D1RA03395F-s830

RA-011-D1RA03395F-s831

RA-011-D1RA03395F-s832

RA-011-D1RA03395F-s833

RA-011-D1RA03395F-s834

RA-011-D1RA03395F-s835

RA-011-D1RA03395F-s836

RA-011-D1RA03395F-s837

RA-011-D1RA03395F-s838

RA-011-D1RA03395F-s839

RA-011-D1RA03395F-s840

RA-011-D1RA03395F-s841

RA-011-D1RA03395F-s842

RA-011-D1RA03395F-s843

RA-011-D1RA03395F-s844

RA-011-D1RA03395F-s845

RA-011-D1RA03395F-s846

RA-011-D1RA03395F-s847

RA-011-D1RA03395F-s848

RA-011-D1RA03395F-s849

RA-011-D1RA03395F-s850

RA-011-D1RA03395F-s851

RA-011-D1RA03395F-s852

RA-011-D1RA03395F-s853

RA-011-D1RA03395F-s854

RA-011-D1RA03395F-s855

RA-011-D1RA03395F-s856

RA-011-D1RA03395F-s857

RA-011-D1RA03395F-s858

RA-011-D1RA03395F-s859

RA-011-D1RA03395F-s860

RA-011-D1RA03395F-s861

RA-011-D1RA03395F-s862

RA-011-D1RA03395F-s863

RA-011-D1RA03395F-s864

RA-011-D1RA03395F-s865

RA-011-D1RA03395F-s866

RA-011-D1RA03395F-s867

RA-011-D1RA03395F-s868

RA-011-D1RA03395F-s869

RA-011-D1RA03395F-s870

RA-011-D1RA03395F-s871

RA-011-D1RA03395F-s872

RA-011-D1RA03395F-s873

RA-011-D1RA03395F-s874

RA-011-D1RA03395F-s875

RA-011-D1RA03395F-s876

RA-011-D1RA03395F-s877

RA-011-D1RA03395F-s878

RA-011-D1RA03395F-s879

RA-011-D1RA03395F-s880

RA-011-D1RA03395F-s881

RA-011-D1RA03395F-s882

RA-011-D1RA03395F-s883

RA-011-D1RA03395F-s884

RA-011-D1RA03395F-s885

RA-011-D1RA03395F-s886

RA-011-D1RA03395F-s887

RA-011-D1RA03395F-s888

RA-011-D1RA03395F-s889

RA-011-D1RA03395F-s890

RA-011-D1RA03395F-s891

RA-011-D1RA03395F-s892

RA-011-D1RA03395F-s893

RA-011-D1RA03395F-s894

RA-011-D1RA03395F-s895

RA-011-D1RA03395F-s896

RA-011-D1RA03395F-s897

RA-011-D1RA03395F-s898

RA-011-D1RA03395F-s899

RA-011-D1RA03395F-s900

RA-011-D1RA03395F-s901

RA-011-D1RA03395F-s902

RA-011-D1RA03395F-s903

RA-011-D1RA03395F-s904

RA-011-D1RA03395F-s905

RA-011-D1RA03395F-s906

RA-011-D1RA03395F-s907

RA-011-D1RA03395F-s908

RA-011-D1RA03395F-s909

RA-011-D1RA03395F-s910

RA-011-D1RA03395F-s911

RA-011-D1RA03395F-s912

RA-011-D1RA03395F-s913

RA-011-D1RA03395F-s914

RA-011-D1RA03395F-s915

RA-011-D1RA03395F-s916

RA-011-D1RA03395F-s917

RA-011-D1RA03395F-s918

RA-011-D1RA03395F-s919

RA-011-D1RA03395F-s920

RA-011-D1RA03395F-s921

RA-011-D1RA03395F-s922

RA-011-D1RA03395F-s923

RA-011-D1RA03395F-s924

RA-011-D1RA03395F-s925

RA-011-D1RA03395F-s926

RA-011-D1RA03395F-s927

RA-011-D1RA03395F-s928

RA-011-D1RA03395F-s929

RA-011-D1RA03395F-s930

RA-011-D1RA03395F-s931

RA-011-D1RA03395F-s932

RA-011-D1RA03395F-s933

RA-011-D1RA03395F-s934

RA-011-D1RA03395F-s935

RA-011-D1RA03395F-s936

RA-011-D1RA03395F-s937

RA-011-D1RA03395F-s938

RA-011-D1RA03395F-s939

RA-011-D1RA03395F-s940

RA-011-D1RA03395F-s941

RA-011-D1RA03395F-s942

RA-011-D1RA03395F-s943

RA-011-D1RA03395F-s944

RA-011-D1RA03395F-s945

RA-011-D1RA03395F-s946

RA-011-D1RA03395F-s947

RA-011-D1RA03395F-s948

RA-011-D1RA03395F-s949

RA-011-D1RA03395F-s950

RA-011-D1RA03395F-s951

RA-011-D1RA03395F-s952

RA-011-D1RA03395F-s953

RA-011-D1RA03395F-s954

RA-011-D1RA03395F-s955

RA-011-D1RA03395F-s956

RA-011-D1RA03395F-s957

RA-011-D1RA03395F-s958

RA-011-D1RA03395F-s959

RA-011-D1RA03395F-s960

RA-011-D1RA03395F-s961

RA-011-D1RA03395F-s962

RA-011-D1RA03395F-s963

RA-011-D1RA03395F-s964

RA-011-D1RA03395F-s965

RA-011-D1RA03395F-s966

RA-011-D1RA03395F-s967

RA-011-D1RA03395F-s968

RA-011-D1RA03395F-s969

RA-011-D1RA03395F-s970

RA-011-D1RA03395F-s971

RA-011-D1RA03395F-s972

RA-011-D1RA03395F-s973

RA-011-D1RA03395F-s974

RA-011-D1RA03395F-s975

RA-011-D1RA03395F-s976

RA-011-D1RA03395F-s977

RA-011-D1RA03395F-s978

RA-011-D1RA03395F-s979

RA-011-D1RA03395F-s980

RA-011-D1RA03395F-s981

RA-011-D1RA03395F-s982

RA-011-D1RA03395F-s983

RA-011-D1RA03395F-s984

RA-011-D1RA03395F-s985

RA-011-D1RA03395F-s986

RA-011-D1RA03395F-s987

RA-011-D1RA03395F-s988

RA-011-D1RA03395F-s989

RA-011-D1RA03395F-s990

RA-011-D1RA03395F-s991

RA-011-D1RA03395F-s992

RA-011-D1RA03395F-s993

RA-011-D1RA03395F-s994

RA-011-D1RA03395F-s995

RA-011-D1RA03395F-s996

RA-011-D1RA03395F-s997

RA-011-D1RA03395F-s998

RA-011-D1RA03395F-s999

RA-011-D1RA03395F-s1000

RA-011-D1RA03395F-s1001

RA-011-D1RA03395F-s1002

RA-011-D1RA03395F-s1003

RA-011-D1RA03395F-s1004

RA-011-D1RA03395F-s1005

RA-011-D1RA03395F-s1006

RA-011-D1RA03395F-s1007

RA-011-D1RA03395F-s1008

RA-011-D1RA03395F-s1009

RA-011-D1RA03395F-s1010

RA-011-D1RA03395F-s1011

RA-011-D1RA03395F-s1012

RA-011-D1RA03395F-s1013

RA-011-D1RA03395F-s1014

RA-011-D1RA03395F-s1015

RA-011-D1RA03395F-s1016

RA-011-D1RA03395F-s1017

RA-011-D1RA03395F-s1018

RA-011-D1RA03395F-s1019

RA-011-D1RA03395F-s1020

RA-011-D1RA03395F-s1021

RA-011-D1RA03395F-s1022

RA-011-D1RA03395F-s1023

RA-011-D1RA03395F-s1024

RA-011-D1RA03395F-s1025

RA-011-D1RA03395F-s1026

RA-011-D1RA03395F-s1027

RA-011-D1RA03395F-s1028

RA-011-D1RA03395F-s1029

RA-011-D1RA03395F-s1030

RA-011-D1RA03395F-s1031

RA-011-D1RA03395F-s1032

RA-011-D1RA03395F-s1033

RA-011-D1RA03395F-s1034

RA-011-D1RA03395F-s1035

RA-011-D1RA03395F-s1036

RA-011-D1RA03395F-s1037

RA-011-D1RA03395F-s1038

RA-011-D1RA03395F-s1039

RA-011-D1RA03395F-s1040

RA-011-D1RA03395F-s1041

RA-011-D1RA03395F-s1042

RA-011-D1RA03395F-s1043

RA-011-D1RA03395F-s1044

RA-011-D1RA03395F-s1045

RA-011-D1RA03395F-s1046

RA-011-D1RA03395F-s1047

RA-011-D1RA03395F-s1048

RA-011-D1RA03395F-s1049

RA-011-D1RA03395F-s1050

RA-011-D1RA03395F-s1051

RA-011-D1RA03395F-s1052

RA-011-D1RA03395F-s1053

RA-011-D1RA03395F-s1054

RA-011-D1RA03395F-s1055

RA-011-D1RA03395F-s1056

RA-011-D1RA03395F-s1057

RA-011-D1RA03395F-s1058

RA-011-D1RA03395F-s1059

RA-011-D1RA03395F-s1060

RA-011-D1RA03395F-s1061

RA-011-D1RA03395F-s1062

RA-011-D1RA03395F-s1063

RA-011-D1RA03395F-s1064

RA-011-D1RA03395F-s1065

RA-011-D1RA03395F-s1066

RA-011-D1RA03395F-s1067

RA-011-D1RA03395F-s1068

RA-011-D1RA03395F-s1069

RA-011-D1RA03395F-s1070

RA-011-D1RA03395F-s1071

RA-011-D1RA03395F-s1072

RA-011-D1RA03395F-s1073

RA-011-D1RA03395F-s1074

RA-011-D1RA03395F-s1075

RA-011-D1RA03395F-s1076

RA-011-D1RA03395F-s1077

RA-011-D1RA03395F-s1078

RA-011-D1RA03395F-s1079

RA-011-D1RA03395F-s1080

RA-011-D1RA03395F-s1081

RA-011-D1RA03395F-s1082

RA-011-D1RA03395F-s1083

RA-011-D1RA03395F-s1084

RA-011-D1RA03395F-s1085

RA-011-D1RA03395F-s1086

RA-011-D1RA03395F-s1087

RA-011-D1RA03395F-s1088

RA-011-D1RA03395F-s1089

RA-011-D1RA03395F-s1090

RA-011-D1RA03395F-s1091

RA-011-D1RA03395F-s1092

RA-011-D1RA03395F-s1093

RA-011-D1RA03395F-s1094

RA-011-D1RA03395F-s1095

RA-011-D1RA03395F-s1096

RA-011-D1RA03395F-s1097

RA-011-D1RA03395F-s1098

RA-011-D1RA03395F-s1099

RA-011-D1RA03395F-s1100

RA-011-D1RA03395F-s1101

RA-011-D1RA03395F-s1102

RA-011-D1RA03395F-s1103

RA-011-D1RA03395F-s1104

RA-011-D1RA03395F-s1105

RA-011-D1RA03395F-s1106

RA-011-D1RA03395F-s1107

RA-011-D1RA03395F-s1108

RA-011-D1RA03395F-s1109

RA-011-D1RA03395F-s1110

RA-011-D1RA03395F-s1111

RA-011-D1RA03395F-s1112

RA-011-D1RA03395F-s1113

RA-011-D1RA03395F-s1114

RA-011-D1RA03395F-s1115

RA-011-D1RA03395F-s1116

RA-011-D1RA03395F-s1117

RA-011-D1RA03395F-s1118

RA-011-D1RA03395F-s1119

RA-011-D1RA03395F-s1120

RA-011-D1RA03395F-s1121

RA-011-D1RA03395F-s1122

RA-011-D1RA03395F-s1123

RA-011-D1RA03395F-s1124

RA-011-D1RA03395F-s1125

RA-011-D1RA03395F-s1126

RA-011-D1RA03395F-s1127

RA-011-D1RA03395F-s1128

RA-011-D1RA03395F-s1129

RA-011-D1RA03395F-s1130

RA-011-D1RA03395F-s1131

RA-011-D1RA03395F-s1132

RA-011-D1RA03395F-s1133

RA-011-D1RA03395F-s1134

RA-011-D1RA03395F-s1135

RA-011-D1RA03395F-s1136

RA-011-D1RA03395F-s1137

RA-011-D1RA03395F-s1138

RA-011-D1RA03395F-s1139

RA-011-D1RA03395F-s1140

RA-011-D1RA03395F-s1141

RA-011-D1RA03395F-s1142

RA-011-D1RA03395F-s1143

RA-011-D1RA03395F-s1144

RA-011-D1RA03395F-s1145

RA-011-D1RA03395F-s1146

RA-011-D1RA03395F-s1147

RA-011-D1RA03395F-s1148

RA-011-D1RA03395F-s1149

RA-011-D1RA03395F-s1150

RA-011-D1RA03395F-s1151

RA-011-D1RA03395F-s1152

RA-011-D1RA03395F-s1153

RA-011-D1RA03395F-s1154

RA-011-D1RA03395F-s1155

RA-011-D1RA03395F-s1156

RA-011-D1RA03395F-s1157

RA-011-D1RA03395F-s1158

RA-011-D1RA03395F-s1159

RA-011-D1RA03395F-s1160

RA-011-D1RA03395F-s1161

RA-011-D1RA03395F-s1162

RA-011-D1RA03395F-s1163

RA-011-D1RA03395F-s1164

RA-011-D1RA03395F-s1165

RA-011-D1RA03395F-s1166

RA-011-D1RA03395F-s1167

RA-011-D1RA03395F-s1168

RA-011-D1RA03395F-s1169

RA-011-D1RA03395F-s1170

RA-011-D1RA03395F-s1171

RA-011-D1RA03395F-s1172

RA-011-D1RA03395F-s1173

RA-011-D1RA03395F-s1174

RA-011-D1RA03395F-s1175

RA-011-D1RA03395F-s1176

RA-011-D1RA03395F-s1177

RA-011-D1RA03395F-s1178

RA-011-D1RA03395F-s1179

RA-011-D1RA03395F-s1180

RA-011-D1RA03395F-s1181

RA-011-D1RA03395F-s1182

RA-011-D1RA03395F-s1183

RA-011-D1RA03395F-s1184

RA-011-D1RA03395F-s1185

RA-011-D1RA03395F-s1186

RA-011-D1RA03395F-s1187

RA-011-D1RA03395F-s1188

RA-011-D1RA03395F-s1189

RA-011-D1RA03395F-s1190

RA-011-D1RA03395F-s1191

RA-011-D1RA03395F-s1192

RA-011-D1RA03395F-s1193

RA-011-D1RA03395F-s1194

RA-011-D1RA03395F-s1195

RA-011-D1RA03395F-s1196

RA-011-D1RA03395F-s1197

RA-011-D1RA03395F-s1198

RA-011-D1RA03395F-s1199

RA-011-D1RA03395F-s1200

RA-011-D1RA03395F-s1201

RA-011-D1RA03395F-s1202

RA-011-D1RA03395F-s1203

RA-011-D1RA03395F-s1204

RA-011-D1RA03395F-s1205

RA-011-D1RA03395F-s1206

RA-011-D1RA03395F-s1207

RA-011-D1RA03395F-s1208

RA-011-D1RA03395F-s1209

RA-011-D1RA03395F-s1210

RA-011-D1RA03395F-s1211

RA-011-D1RA03395F-s1212

RA-011-D1RA03395F-s1213

RA-011-D1RA03395F-s1214

RA-011-D1RA03395F-s1215

RA-011-D1RA03395F-s1216

RA-011-D1RA03395F-s1217

RA-011-D1RA03395F-s1218

RA-011-D1RA03395F-s1219

RA-011-D1RA03395F-s1220

RA-011-D1RA03395F-s1221

RA-011-D1RA03395F-s1222

RA-011-D1RA03395F-s1223

RA-011-D1RA03395F-s1224

RA-011-D1RA03395F-s1225

RA-011-D1RA03395F-s1226

RA-011-D1RA03395F-s1227

RA-011-D1RA03395F-s1228

RA-011-D1RA03395F-s1229

RA-011-D1RA03395F-s1230

RA-011-D1RA03395F-s1231

RA-011-D1RA03395F-s1232

RA-011-D1RA03395F-s1233

RA-011-D1RA03395F-s1234

RA-011-D1RA03395F-s1235

RA-011-D1RA03395F-s1236

RA-011-D1RA03395F-s1237

RA-011-D1RA03395F-s1238

RA-011-D1RA03395F-s1239

RA-011-D1RA03395F-s1240

RA-011-D1RA03395F-s1241

RA-011-D1RA03395F-s1242

RA-011-D1RA03395F-s1243

RA-011-D1RA03395F-s1244

RA-011-D1RA03395F-s1245

RA-011-D1RA03395F-s1246

RA-011-D1RA03395F-s1247

RA-011-D1RA03395F-s1248

RA-011-D1RA03395F-s1249

RA-011-D1RA03395F-s1250

RA-011-D1RA03395F-s1251

RA-011-D1RA03395F-s1252

RA-011-D1RA03395F-s1253

RA-011-D1RA03395F-s1254

RA-011-D1RA03395F-s1255

RA-011-D1RA03395F-s1256

RA-011-D1RA03395F-s1257

RA-011-D1RA03395F-s1258

RA-011-D1RA03395F-s1259

RA-011-D1RA03395F-s1260

RA-011-D1RA03395F-s1261

RA-011-D1RA03395F-s1262

RA-011-D1RA03395F-s1263

RA-011-D1RA03395F-s1264

RA-011-D1RA03395F-s1265

RA-011-D1RA03395F-s1266

RA-011-D1RA03395F-s1267

RA-011-D1RA03395F-s1268

RA-011-D1RA03395F-s1269

RA-011-D1RA03395F-s1270

RA-011-D1RA03395F-s1271

RA-011-D1RA03395F-s1272

RA-011-D1RA03395F-s1273

RA-011-D1RA03395F-s1274

RA-011-D1RA03395F-s1275

RA-011-D1RA03395F-s1276

RA-011-D1RA03395F-s1277

RA-011-D1RA03395F-s1278

RA-011-D1RA03395F-s1279

RA-011-D1RA03395F-s1280

RA-011-D1RA03395F-s1281

RA-011-D1RA03395F-s1282

RA-011-D1RA03395F-s1283

RA-011-D1RA03395F-s1284

RA-011-D1RA03395F-s1285

RA-011-D1RA03395F-s1286

RA-011-D1RA03395F-s1287

RA-011-D1RA03395F-s1288

RA-011-D1RA03395F-s1289

RA-011-D1RA03395F-s1290

RA-011-D1RA03395F-s1291

RA-011-D1RA03395F-s1292

RA-011-D1RA03395F-s1293

RA-011-D1RA03395F-s1294

RA-011-D1RA03395F-s1295

RA-011-D1RA03395F-s1296

RA-011-D1RA03395F-s1297

RA-011-D1RA03395F-s1298

RA-011-D1RA03395F-s1299

RA-011-D1RA03395F-s1300

RA-011-D1RA03395F-s1301

RA-011-D1RA03395F-s1302

RA-011-D1RA03395F-s1303

RA-011-D1RA03395F-s1304

RA-011-D1RA03395F-s1305

RA-011-D1RA03395F-s1306

RA-011-D1RA03395F-s1307

RA-011-D1RA03395F-s1308

RA-011-D1RA03395F-s1309

RA-011-D1RA03395F-s1310

RA-011-D1RA03395F-s1311

RA-011-D1RA03395F-s1312

RA-011-D1RA03395F-s1313

RA-011-D1RA03395F-s1314

RA-011-D1RA03395F-s1315

RA-011-D1RA03395F-s1316

RA-011-D1RA03395F-s1317

RA-011-D1RA03395F-s1318

RA-011-D1RA03395F-s1319

RA-011-D1RA03395F-s1320

RA-011-D1RA03395F-s1321

RA-011-D1RA03395F-s1322

RA-011-D1RA03395F-s1323

RA-011-D1RA03395F-s1324

RA-011-D1RA03395F-s1325

RA-011-D1RA03395F-s1326

RA-011-D1RA03395F-s1327

RA-011-D1RA03395F-s1328

RA-011-D1RA03395F-s1329

RA-011-D1RA03395F-s1330

RA-011-D1RA03395F-s1331

RA-011-D1RA03395F-s1332

RA-011-D1RA03395F-s1333

RA-011-D1RA03395F-s1334

RA-011-D1RA03395F-s1335

RA-011-D1RA03395F-s1336

RA-011-D1RA03395F-s1337

RA-011-D1RA03395F-s1338

RA-011-D1RA03395F-s1339

RA-011-D1RA03395F-s1340

RA-011-D1RA03395F-s1341

RA-011-D1RA03395F-s1342

RA-011-D1RA03395F-s1343

RA-011-D1RA03395F-s1344

RA-011-D1RA03395F-s1345

RA-011-D1RA03395F-s1346

RA-011-D1RA03395F-s1347

RA-011-D1RA03395F-s1348

RA-011-D1RA03395F-s1349

RA-011-D1RA03395F-s1350

RA-011-D1RA03395F-s1351

RA-011-D1RA03395F-s1352

RA-011-D1RA03395F-s1353

RA-011-D1RA03395F-s1354

RA-011-D1RA03395F-s1355

RA-011-D1RA03395F-s1356

RA-011-D1RA03395F-s1357

RA-011-D1RA03395F-s1358

RA-011-D1RA03395F-s1359

RA-011-D1RA03395F-s1360

RA-011-D1RA03395F-s1361

RA-011-D1RA03395F-s1362

RA-011-D1RA03395F-s1363

RA-011-D1RA03395F-s1364

RA-011-D1RA03395F-s1365

RA-011-D1RA03395F-s1366

RA-011-D1RA03395F-s1367

RA-011-D1RA03395F-s1368

RA-011-D1RA03395F-s1369

RA-011-D1RA03395F-s1370

RA-011-D1RA03395F-s1371

RA-011-D1RA03395F-s1372

RA-011-D1RA03395F-s1373

RA-011-D1RA03395F-s1374

RA-011-D1RA03395F-s1375

RA-011-D1RA03395F-s1376

RA-011-D1RA03395F-s1377

RA-011-D1RA03395F-s1378

RA-011-D1RA03395F-s1379

RA-011-D1RA03395F-s1380

RA-011-D1RA03395F-s1381

RA-011-D1RA03395F-s1382

RA-011-D1RA03395F-s1383

RA-011-D1RA03395F-s1384

RA-011-D1RA03395F-s1385

RA-011-D1RA03395F-s1386

RA-011-D1RA03395F-s1387

RA-011-D1RA03395F-s1388

RA-011-D1RA03395F-s1389

RA-011-D1RA03395F-s1390

RA-011-D1RA03395F-s1391

RA-011-D1RA03395F-s1392

RA-011-D1RA03395F-s1393

RA-011-D1RA03395F-s1394

RA-011-D1RA03395F-s1395

RA-011-D1RA03395F-s1396

RA-011-D1RA03395F-s1397

RA-011-D1RA03395F-s1398

RA-011-D1RA03395F-s1399

RA-011-D1RA03395F-s1400

RA-011-D1RA03395F-s1401

RA-011-D1RA03395F-s1402

RA-011-D1RA03395F-s1403

RA-011-D1RA03395F-s1404

RA-011-D1RA03395F-s1405

RA-011-D1RA03395F-s1406

RA-011-D1RA03395F-s1407

RA-011-D1RA03395F-s1408

RA-011-D1RA03395F-s1409

RA-011-D1RA03395F-s1410

RA-011-D1RA03395F-s1411

RA-011-D1RA03395F-s1412

RA-011-D1RA03395F-s1413

RA-011-D1RA03395F-s1414

RA-011-D1RA03395F-s1415

RA-011-D1RA03395F-s1416

RA-011-D1RA03395F-s1417

RA-011-D1RA03395F-s1418

RA-011-D1RA03395F-s1419

RA-011-D1RA03395F-s1420

RA-011-D1RA03395F-s1421

RA-011-D1RA03395F-s1422

RA-011-D1RA03395F-s1423

RA-011-D1RA03395F-s1424

RA-011-D1RA03395F-s1425

RA-011-D1RA03395F-s1426

RA-011-D1RA03395F-s1427

RA-011-D1RA03395F-s1428

RA-011-D1RA03395F-s1429

RA-011-D1RA03395F-s1430

RA-011-D1RA03395F-s1431

RA-011-D1RA03395F-s1432

RA-011-D1RA03395F-s1433

RA-011-D1RA03395F-s1434

RA-011-D1RA03395F-s1435

RA-011-D1RA03395F-s1436

RA-011-D1RA03395F-s1437

RA-011-D1RA03395F-s1438

RA-011-D1RA03395F-s1439

RA-011-D1RA03395F-s1440

RA-011-D1RA03395F-s1441

RA-011-D1RA03395F-s1442

RA-011-D1RA03395F-s1443

RA-011-D1RA03395F-s1444

RA-011-D1RA03395F-s1445

RA-011-D1RA03395F-s1446

RA-011-D1RA03395F-s1447

RA-011-D1RA03395F-s1448

RA-011-D1RA03395F-s1449

RA-011-D1RA03395F-s1450

RA-011-D1RA03395F-s1451

RA-011-D1RA03395F-s1452

RA-011-D1RA03395F-s1453

RA-011-D1RA03395F-s1454

RA-011-D1RA03395F-s1455

RA-011-D1RA03395F-s1456

RA-011-D1RA03395F-s1457

RA-011-D1RA03395F-s1458

RA-011-D1RA03395F-s1459

RA-011-D1RA03395F-s1460

RA-011-D1RA03395F-s1461

RA-011-D1RA03395F-s1462

RA-011-D1RA03395F-s1463

RA-011-D1RA03395F-s1464

RA-011-D1RA03395F-s1465

RA-011-D1RA03395F-s1466

RA-011-D1RA03395F-s1467

RA-011-D1RA03395F-s1468

RA-011-D1RA03395F-s1469

RA-011-D1RA03395F-s1470

RA-011-D1RA03395F-s1471

RA-011-D1RA03395F-s1472

RA-011-D1RA03395F-s1473

RA-011-D1RA03395F-s1474

RA-011-D1RA03395F-s1475

RA-011-D1RA03395F-s1476

RA-011-D1RA03395F-s1477

RA-011-D1RA03395F-s1478

RA-011-D1RA03395F-s1479

RA-011-D1RA03395F-s1480

RA-011-D1RA03395F-s1481

RA-011-D1RA03395F-s1482

RA-011-D1RA03395F-s1483

RA-011-D1RA03395F-s1484

RA-011-D1RA03395F-s1485

RA-011-D1RA03395F-s1486

RA-011-D1RA03395F-s1487

RA-011-D1RA03395F-s1488

RA-011-D1RA03395F-s1489

RA-011-D1RA03395F-s1490

RA-011-D1RA03395F-s1491

RA-011-D1RA03395F-s1492

RA-011-D1RA03395F-s1493

RA-011-D1RA03395F-s1494

RA-011-D1RA03395F-s1495

RA-011-D1RA03395F-s1496

RA-011-D1RA03395F-s1497

RA-011-D1RA03395F-s1498

RA-011-D1RA03395F-s1499

RA-011-D1RA03395F-s1500

RA-011-D1RA03395F-s1501

RA-011-D1RA03395F-s1502

RA-011-D1RA03395F-s1503

RA-011-D1RA03395F-s1504

RA-011-D1RA03395F-s1505

RA-011-D1RA03395F-s1506

RA-011-D1RA03395F-s1507

RA-011-D1RA03395F-s1508

RA-011-D1RA03395F-s1509

RA-011-D1RA03395F-s1510

RA-011-D1RA03395F-s1511

RA-011-D1RA03395F-s1512

RA-011-D1RA03395F-s1513

RA-011-D1RA03395F-s1514

RA-011-D1RA03395F-s1515

RA-011-D1RA03395F-s1516

RA-011-D1RA03395F-s1517

RA-011-D1RA03395F-s1518

RA-011-D1RA03395F-s1519

RA-011-D1RA03395F-s1520

RA-011-D1RA03395F-s1521

RA-011-D1RA03395F-s1522

RA-011-D1RA03395F-s1523

RA-011-D1RA03395F-s1524

RA-011-D1RA03395F-s1525

RA-011-D1RA03395F-s1526

RA-011-D1RA03395F-s1527

RA-011-D1RA03395F-s1528

RA-011-D1RA03395F-s1529

RA-011-D1RA03395F-s1530

RA-011-D1RA03395F-s1531

RA-011-D1RA03395F-s1532

RA-011-D1RA03395F-s1533

RA-011-D1RA03395F-s1534

RA-011-D1RA03395F-s1535

RA-011-D1RA03395F-s1536

RA-011-D1RA03395F-s1537

RA-011-D1RA03395F-s1538

RA-011-D1RA03395F-s1539

RA-011-D1RA03395F-s1540

RA-011-D1RA03395F-s1541

RA-011-D1RA03395F-s1542

RA-011-D1RA03395F-s1543

RA-011-D1RA03395F-s1544

RA-011-D1RA03395F-s1545

RA-011-D1RA03395F-s1546

RA-011-D1RA03395F-s1547

RA-011-D1RA03395F-s1548

RA-011-D1RA03395F-s1549

RA-011-D1RA03395F-s1550

RA-011-D1RA03395F-s1551

RA-011-D1RA03395F-s1552

RA-011-D1RA03395F-s1553

RA-011-D1RA03395F-s1554

RA-011-D1RA03395F-s1555

RA-011-D1RA03395F-s1556

RA-011-D1RA03395F-s1557

RA-011-D1RA03395F-s1558

RA-011-D1RA03395F-s1559

RA-011-D1RA03395F-s1560

RA-011-D1RA03395F-s1561

RA-011-D1RA03395F-s1562

RA-011-D1RA03395F-s1563

RA-011-D1RA03395F-s1564

RA-011-D1RA03395F-s1565

RA-011-D1RA03395F-s1566

RA-011-D1RA03395F-s1567

RA-011-D1RA03395F-s1568

RA-011-D1RA03395F-s1569

RA-011-D1RA03395F-s1570

RA-011-D1RA03395F-s1571

RA-011-D1RA03395F-s1572

RA-011-D1RA03395F-s1573

RA-011-D1RA03395F-s1574

RA-011-D1RA03395F-s1575

RA-011-D1RA03395F-s1576

RA-011-D1RA03395F-s1577

RA-011-D1RA03395F-s1578

RA-011-D1RA03395F-s1579

RA-011-D1RA03395F-s1580

RA-011-D1RA03395F-s1581

RA-011-D1RA03395F-s1582

RA-011-D1RA03395F-s1583

RA-011-D1RA03395F-s1584

RA-011-D1RA03395F-s1585

RA-011-D1RA03395F-s1586

RA-011-D1RA03395F-s1587

RA-011-D1RA03395F-s1588

RA-011-D1RA03395F-s1589

RA-011-D1RA03395F-s1590

RA-011-D1RA03395F-s1591

RA-011-D1RA03395F-s1592

RA-011-D1RA03395F-s1593

RA-011-D1RA03395F-s1594

RA-011-D1RA03395F-s1595

RA-011-D1RA03395F-s1596

RA-011-D1RA03395F-s1597

RA-011-D1RA03395F-s1598

RA-011-D1RA03395F-s1599

RA-011-D1RA03395F-s1600

RA-011-D1RA03395F-s1601

RA-011-D1RA03395F-s1602

RA-011-D1RA03395F-s1603

RA-011-D1RA03395F-s1604

RA-011-D1RA03395F-s1605

RA-011-D1RA03395F-s1606

RA-011-D1RA03395F-s1607

RA-011-D1RA03395F-s1608

RA-011-D1RA03395F-s1609

RA-011-D1RA03395F-s1610

RA-011-D1RA03395F-s1611

RA-011-D1RA03395F-s1612

RA-011-D1RA03395F-s1613

RA-011-D1RA03395F-s1614

RA-011-D1RA03395F-s1615

RA-011-D1RA03395F-s1616

RA-011-D1RA03395F-s1617

RA-011-D1RA03395F-s1618

RA-011-D1RA03395F-s1619

RA-011-D1RA03395F-s1620

RA-011-D1RA03395F-s1621

RA-011-D1RA03395F-s1622

RA-011-D1RA03395F-s1623

RA-011-D1RA03395F-s1624

RA-011-D1RA03395F-s1625

RA-011-D1RA03395F-s1626

RA-011-D1RA03395F-s1627

RA-011-D1RA03395F-s1628

RA-011-D1RA03395F-s1629

RA-011-D1RA03395F-s1630

RA-011-D1RA03395F-s1631

RA-011-D1RA03395F-s1632

RA-011-D1RA03395F-s1633

RA-011-D1RA03395F-s1634

RA-011-D1RA03395F-s1635

RA-011-D1RA03395F-s1636

RA-011-D1RA03395F-s1637

RA-011-D1RA03395F-s1638

RA-011-D1RA03395F-s1639

RA-011-D1RA03395F-s1640

RA-011-D1RA03395F-s1641

RA-011-D1RA03395F-s1642

RA-011-D1RA03395F-s1643

RA-011-D1RA03395F-s1644

RA-011-D1RA03395F-s1645

RA-011-D1RA03395F-s1646

RA-011-D1RA03395F-s1647

RA-011-D1RA03395F-s1648

RA-011-D1RA03395F-s1649

RA-011-D1RA03395F-s1650

RA-011-D1RA03395F-s1651

RA-011-D1RA03395F-s1652

RA-011-D1RA03395F-s1653

RA-011-D1RA03395F-s1654

RA-011-D1RA03395F-s1655

RA-011-D1RA03395F-s1656

RA-011-D1RA03395F-s1657

RA-011-D1RA03395F-s1658

RA-011-D1RA03395F-s1659

RA-011-D1RA03395F-s1660

RA-011-D1RA03395F-s1661

RA-011-D1RA03395F-s1662

RA-011-D1RA03395F-s1663

RA-011-D1RA03395F-s1664

RA-011-D1RA03395F-s1665

RA-011-D1RA03395F-s1666

RA-011-D1RA03395F-s1667

RA-011-D1RA03395F-s1668

RA-011-D1RA03395F-s1669

RA-011-D1RA03395F-s1670

RA-011-D1RA03395F-s1671

RA-011-D1RA03395F-s1672

RA-011-D1RA03395F-s1673

RA-011-D1RA03395F-s1674

RA-011-D1RA03395F-s1675

RA-011-D1RA03395F-s1676

RA-011-D1RA03395F-s1677

RA-011-D1RA03395F-s1678

RA-011-D1RA03395F-s1679

RA-011-D1RA03395F-s1680

RA-011-D1RA03395F-s1681

RA-011-D1RA03395F-s1682

RA-011-D1RA03395F-s1683

RA-011-D1RA03395F-s1684

RA-011-D1RA03395F-s1685

RA-011-D1RA03395F-s1686

RA-011-D1RA03395F-s1687

RA-011-D1RA03395F-s1688

RA-011-D1RA03395F-s1689

RA-011-D1RA03395F-s1690

RA-011-D1RA03395F-s1691

RA-011-D1RA03395F-s1692

RA-011-D1RA03395F-s1693

RA-011-D1RA03395F-s1694

RA-011-D1RA03395F-s1695

RA-011-D1RA03395F-s1696

RA-011-D1RA03395F-s1697

RA-011-D1RA03395F-s1698

RA-011-D1RA03395F-s1699

RA-011-D1RA03395F-s1700

RA-011-D1RA03395F-s1701

RA-011-D1RA03395F-s1702

RA-011-D1RA03395F-s1703

RA-011-D1RA03395F-s1704

RA-011-D1RA03395F-s1705

RA-011-D1RA03395F-s1706

RA-011-D1RA03395F-s1707

RA-011-D1RA03395F-s1708

RA-011-D1RA03395F-s1709

RA-011-D1RA03395F-s1710

RA-011-D1RA03395F-s1711

RA-011-D1RA03395F-s1712

RA-011-D1RA03395F-s1713

RA-011-D1RA03395F-s1714

RA-011-D1RA03395F-s1715

RA-011-D1RA03395F-s1716

RA-011-D1RA03395F-s1717

RA-011-D1RA03395F-s1718

RA-011-D1RA03395F-s1719

RA-011-D1RA03395F-s1720

RA-011-D1RA03395F-s1721

RA-011-D1RA03395F-s1722

RA-011-D1RA03395F-s1723

RA-011-D1RA03395F-s1724

RA-011-D1RA03395F-s1725

RA-011-D1RA03395F-s1726

RA-011-D1RA03395F-s1727

RA-011-D1RA03395F-s1728

RA-011-D1RA03395F-s1729

RA-011-D1RA03395F-s1730

RA-011-D1RA03395F-s1731

RA-011-D1RA03395F-s1732

RA-011-D1RA03395F-s1733

RA-011-D1RA03395F-s1734

RA-011-D1RA03395F-s1735

RA-011-D1RA03395F-s1736

RA-011-D1RA03395F-s1737

RA-011-D1RA03395F-s1738

RA-011-D1RA03395F-s1739

RA-011-D1RA03395F-s1740

RA-011-D1RA03395F-s1741

RA-011-D1RA03395F-s1742

RA-011-D1RA03395F-s1743

RA-011-D1RA03395F-s1744

RA-011-D1RA03395F-s1745

RA-011-D1RA03395F-s1746

RA-011-D1RA03395F-s1747

RA-011-D1RA03395F-s1748

RA-011-D1RA03395F-s1749

RA-011-D1RA03395F-s1750

RA-011-D1RA03395F-s1751

RA-011-D1RA03395F-s1752

RA-011-D1RA03395F-s1753

RA-011-D1RA03395F-s1754

RA-011-D1RA03395F-s1755

RA-011-D1RA03395F-s1756

RA-011-D1RA03395F-s1757

RA-011-D1RA03395F-s1758

RA-011-D1RA03395F-s1759

RA-011-D1RA03395F-s1760

RA-011-D1RA03395F-s1761

RA-011-D1RA03395F-s1762

RA-011-D1RA03395F-s1763

RA-011-D1RA03395F-s1764

RA-011-D1RA03395F-s1765

RA-011-D1RA03395F-s1766

RA-011-D1RA03395F-s1767

RA-011-D1RA03395F-s1768

RA-011-D1RA03395F-s1769

RA-011-D1RA03395F-s1770

RA-011-D1RA03395F-s1771

RA-011-D1RA03395F-s1772

RA-011-D1RA03395F-s1773

RA-011-D1RA03395F-s1774

RA-011-D1RA03395F-s1775

RA-011-D1RA03395F-s1776

RA-011-D1RA03395F-s1777

RA-011-D1RA03395F-s1778

RA-011-D1RA03395F-s1779

RA-011-D1RA03395F-s1780

RA-011-D1RA03395F-s1781

RA-011-D1RA03395F-s1782

RA-011-D1RA03395F-s1783

RA-011-D1RA03395F-s1784

RA-011-D1RA03395F-s1785

RA-011-D1RA03395F-s1786

RA-011-D1RA03395F-s1787

RA-011-D1RA03395F-s1788

RA-011-D1RA03395F-s1789

RA-011-D1RA03395F-s1790

RA-011-D1RA03395F-s1791

RA-011-D1RA03395F-s1792

RA-011-D1RA03395F-s1793

RA-011-D1RA03395F-s1794

RA-011-D1RA03395F-s1795

RA-011-D1RA03395F-s1796

RA-011-D1RA03395F-s1797

RA-011-D1RA03395F-s1798

RA-011-D1RA03395F-s1799

RA-011-D1RA03395F-s1800

RA-011-D1RA03395F-s1801

RA-011-D1RA03395F-s1802

RA-011-D1RA03395F-s1803

RA-011-D1RA03395F-s1804

RA-011-D1RA03395F-s1805

RA-011-D1RA03395F-s1806

RA-011-D1RA03395F-s1807

RA-011-D1RA03395F-s1808

RA-011-D1RA03395F-s1809

RA-011-D1RA03395F-s1810

RA-011-D1RA03395F-s1811

RA-011-D1RA03395F-s1812

RA-011-D1RA03395F-s1813

RA-011-D1RA03395F-s1814

RA-011-D1RA03395F-s1815

RA-011-D1RA03395F-s1816

RA-011-D1RA03395F-s1817

RA-011-D1RA03395F-s1818

RA-011-D1RA03395F-s1819

RA-011-D1RA03395F-s1820

RA-011-D1RA03395F-s1821

RA-011-D1RA03395F-s1822

RA-011-D1RA03395F-s1823

RA-011-D1RA03395F-s1824

RA-011-D1RA03395F-s1825

RA-011-D1RA03395F-s1826

RA-011-D1RA03395F-s1827

RA-011-D1RA03395F-s1828

RA-011-D1RA03395F-s1829

RA-011-D1RA03395F-s1830

RA-011-D1RA03395F-s1831

RA-011-D1RA03395F-s1832

RA-011-D1RA03395F-s1833

RA-011-D1RA03395F-s1834

RA-011-D1RA03395F-s1835

RA-011-D1RA03395F-s1836

RA-011-D1RA03395F-s1837

RA-011-D1RA03395F-s1838

RA-011-D1RA03395F-s1839

RA-011-D1RA03395F-s1840

RA-011-D1RA03395F-s1841

RA-011-D1RA03395F-s1842

RA-011-D1RA03395F-s1843

RA-011-D1RA03395F-s1844

RA-011-D1RA03395F-s1845

RA-011-D1RA03395F-s1846

RA-011-D1RA03395F-s1847

RA-011-D1RA03395F-s1848

RA-011-D1RA03395F-s1849

RA-011-D1RA03395F-s1850

RA-011-D1RA03395F-s1851

RA-011-D1RA03395F-s1852

RA-011-D1RA03395F-s1853

RA-011-D1RA03395F-s1854

RA-011-D1RA03395F-s1855

RA-011-D1RA03395F-s1856

RA-011-D1RA03395F-s1857

RA-011-D1RA03395F-s1858

RA-011-D1RA03395F-s1859

RA-011-D1RA03395F-s1860

RA-011-D1RA03395F-s1861

RA-011-D1RA03395F-s1862

RA-011-D1RA03395F-s1863

RA-011-D1RA03395F-s1864

RA-011-D1RA03395F-s1865

RA-011-D1RA03395F-s1866

RA-011-D1RA03395F-s1867

RA-011-D1RA03395F-s1868

RA-011-D1RA03395F-s1869

RA-011-D1RA03395F-s1870

RA-011-D1RA03395F-s1871

RA-011-D1RA03395F-s1872

RA-011-D1RA03395F-s1873

RA-011-D1RA03395F-s1874

RA-011-D1RA03395F-s1875

RA-011-D1RA03395F-s1876

RA-011-D1RA03395F-s1877

RA-011-D1RA03395F-s1878

RA-011-D1RA03395F-s1879

RA-011-D1RA03395F-s1880

RA-011-D1RA03395F-s1881

RA-011-D1RA03395F-s1882

RA-011-D1RA03395F-s1883

RA-011-D1RA03395F-s1884

RA-011-D1RA03395F-s1885

RA-011-D1RA03395F-s1886

RA-011-D1RA03395F-s1887

RA-011-D1RA03395F-s1888

RA-011-D1RA03395F-s1889

RA-011-D1RA03395F-s1890

RA-011-D1RA03395F-s1891

RA-011-D1RA03395F-s1892

RA-011-D1RA03395F-s1893

RA-011-D1RA03395F-s1894

RA-011-D1RA03395F-s1895

RA-011-D1RA03395F-s1896

RA-011-D1RA03395F-s1897

RA-011-D1RA03395F-s1898

RA-011-D1RA03395F-s1899

RA-011-D1RA03395F-s1900

RA-011-D1RA03395F-s1901

RA-011-D1RA03395F-s1902

RA-011-D1RA03395F-s1903

RA-011-D1RA03395F-s1904

RA-011-D1RA03395F-s1905

RA-011-D1RA03395F-s1906

RA-011-D1RA03395F-s1907

RA-011-D1RA03395F-s1908

RA-011-D1RA03395F-s1909

RA-011-D1RA03395F-s1910

RA-011-D1RA03395F-s1911

RA-011-D1RA03395F-s1912

RA-011-D1RA03395F-s1913

RA-011-D1RA03395F-s1914

RA-011-D1RA03395F-s1915

RA-011-D1RA03395F-s1916

RA-011-D1RA03395F-s1917

RA-011-D1RA03395F-s1918

RA-011-D1RA03395F-s1919

RA-011-D1RA03395F-s1920

RA-011-D1RA03395F-s1921

RA-011-D1RA03395F-s1922

RA-011-D1RA03395F-s1923

RA-011-D1RA03395F-s1924

RA-011-D1RA03395F-s1925

RA-011-D1RA03395F-s1926

RA-011-D1RA03395F-s1927

RA-011-D1RA03395F-s1928

RA-011-D1RA03395F-s1929

RA-011-D1RA03395F-s1930

RA-011-D1RA03395F-s1931

RA-011-D1RA03395F-s1932

RA-011-D1RA03395F-s1933

RA-011-D1RA03395F-s1934

RA-011-D1RA03395F-s1935

RA-011-D1RA03395F-s1936

RA-011-D1RA03395F-s1937

RA-011-D1RA03395F-s1938

RA-011-D1RA03395F-s1939

RA-011-D1RA03395F-s1940

RA-011-D1RA03395F-s1941

RA-011-D1RA03395F-s1942

RA-011-D1RA03395F-s1943

RA-011-D1RA03395F-s1944

RA-011-D1RA03395F-s1945

RA-011-D1RA03395F-s1946

RA-011-D1RA03395F-s1947

RA-011-D1RA03395F-s1948

RA-011-D1RA03395F-s1949

RA-011-D1RA03395F-s1950

RA-011-D1RA03395F-s1951

RA-011-D1RA03395F-s1952

RA-011-D1RA03395F-s1953

RA-011-D1RA03395F-s1954

RA-011-D1RA03395F-s1955

RA-011-D1RA03395F-s1956

RA-011-D1RA03395F-s1957

RA-011-D1RA03395F-s1958

RA-011-D1RA03395F-s1959

RA-011-D1RA03395F-s1960

RA-011-D1RA03395F-s1961

RA-011-D1RA03395F-s1962

RA-011-D1RA03395F-s1963

RA-011-D1RA03395F-s1964

RA-011-D1RA03395F-s1965

RA-011-D1RA03395F-s1966

RA-011-D1RA03395F-s1967

RA-011-D1RA03395F-s1968

RA-011-D1RA03395F-s1969

RA-011-D1RA03395F-s1970

RA-011-D1RA03395F-s1971

RA-011-D1RA03395F-s1972

RA-011-D1RA03395F-s1973

RA-011-D1RA03395F-s1974

RA-011-D1RA03395F-s1975

RA-011-D1RA03395F-s1976

RA-011-D1RA03395F-s1977

RA-011-D1RA03395F-s1978

RA-011-D1RA03395F-s1979

RA-011-D1RA03395F-s1980

RA-011-D1RA03395F-s1981

RA-011-D1RA03395F-s1982

RA-011-D1RA03395F-s1983

RA-011-D1RA03395F-s1984

RA-011-D1RA03395F-s1985

RA-011-D1RA03395F-s1986

RA-011-D1RA03395F-s1987

RA-011-D1RA03395F-s1988

RA-011-D1RA03395F-s1989

RA-011-D1RA03395F-s1990

RA-011-D1RA03395F-s1991

RA-011-D1RA03395F-s1992

RA-011-D1RA03395F-s1993

RA-011-D1RA03395F-s1994

RA-011-D1RA03395F-s1995

RA-011-D1RA03395F-s1996

RA-011-D1RA03395F-s1997

RA-011-D1RA03395F-s1998

RA-011-D1RA03395F-s1999

RA-011-D1RA03395F-s2000

RA-011-D1RA03395F-s2001

RA-011-D1RA03395F-s2002

RA-011-D1RA03395F-s2003

RA-011-D1RA03395F-s2004

RA-011-D1RA03395F-s2005

RA-011-D1RA03395F-s2006

RA-011-D1RA03395F-s2007

RA-011-D1RA03395F-s2008

RA-011-D1RA03395F-s2009

RA-011-D1RA03395F-s2010

RA-011-D1RA03395F-s2011

RA-011-D1RA03395F-s2012

RA-011-D1RA03395F-s2013

RA-011-D1RA03395F-s2014

RA-011-D1RA03395F-s2015

RA-011-D1RA03395F-s2016

RA-011-D1RA03395F-s2017

RA-011-D1RA03395F-s2018

RA-011-D1RA03395F-s2019

RA-011-D1RA03395F-s2020

RA-011-D1RA03395F-s2021

RA-011-D1RA03395F-s2022

RA-011-D1RA03395F-s2023

RA-011-D1RA03395F-s2024

RA-011-D1RA03395F-s2025

RA-011-D1RA03395F-s2026

RA-011-D1RA03395F-s2027

RA-011-D1RA03395F-s2028

RA-011-D1RA03395F-s2029

RA-011-D1RA03395F-s2030

RA-011-D1RA03395F-s2031

RA-011-D1RA03395F-s2032

RA-011-D1RA03395F-s2033

RA-011-D1RA03395F-s2034

RA-011-D1RA03395F-s2035

RA-011-D1RA03395F-s2036

RA-011-D1RA03395F-s2037

RA-011-D1RA03395F-s2038

RA-011-D1RA03395F-s2039

RA-011-D1RA03395F-s2040

RA-011-D1RA03395F-s2041

RA-011-D1RA03395F-s2042

RA-011-D1RA03395F-s2043

RA-011-D1RA03395F-s2044

RA-011-D1RA03395F-s2045

RA-011-D1RA03395F-s2046

RA-011-D1RA03395F-s2047

RA-011-D1RA03395F-s2048

RA-011-D1RA03395F-s2049

RA-011-D1RA03395F-s2050

RA-011-D1RA03395F-s2051

RA-011-D1RA03395F-s2052

RA-011-D1RA03395F-s2053

RA-011-D1RA03395F-s2054

RA-011-D1RA03395F-s2055

RA-011-D1RA03395F-s2056

RA-011-D1RA03395F-s2057

RA-011-D1RA03395F-s2058

RA-011-D1RA03395F-s2059

RA-011-D1RA03395F-s2060

RA-011-D1RA03395F-s2061

RA-011-D1RA03395F-s2062

RA-011-D1RA03395F-s2063

RA-011-D1RA03395F-s2064

RA-011-D1RA03395F-s2065

RA-011-D1RA03395F-s2066

RA-011-D1RA03395F-s2067

RA-011-D1RA03395F-s2068

RA-011-D1RA03395F-s2069

RA-011-D1RA03395F-s2070

RA-011-D1RA03395F-s2071

RA-011-D1RA03395F-s2072

RA-011-D1RA03395F-s2073

RA-011-D1RA03395F-s2074

RA-011-D1RA03395F-s2075

RA-011-D1RA03395F-s2076

RA-011-D1RA03395F-s2077

RA-011-D1RA03395F-s2078

RA-011-D1RA03395F-s2079

RA-011-D1RA03395F-s2080

RA-011-D1RA03395F-s2081

RA-011-D1RA03395F-s2082

RA-011-D1RA03395F-s2083

RA-011-D1RA03395F-s2084

RA-011-D1RA03395F-s2085

RA-011-D1RA03395F-s2086

RA-011-D1RA03395F-s2087

RA-011-D1RA03395F-s2088

RA-011-D1RA03395F-s2089

RA-011-D1RA03395F-s2090

RA-011-D1RA03395F-s2091

RA-011-D1RA03395F-s2092

RA-011-D1RA03395F-s2093

RA-011-D1RA03395F-s2094

RA-011-D1RA03395F-s2095

RA-011-D1RA03395F-s2096

RA-011-D1RA03395F-s2097

RA-011-D1RA03395F-s2098

RA-011-D1RA03395F-s2099

RA-011-D1RA03395F-s2100

RA-011-D1RA03395F-s2101

RA-011-D1RA03395F-s2102

RA-011-D1RA03395F-s2103

RA-011-D1RA03395F-s2104

RA-011-D1RA03395F-s2105

RA-011-D1RA03395F-s2106

RA-011-D1RA03395F-s2107

RA-011-D1RA03395F-s2108

RA-011-D1RA03395F-s2109

RA-011-D1RA03395F-s2110

RA-011-D1RA03395F-s2111

RA-011-D1RA03395F-s2112

RA-011-D1RA03395F-s2113

RA-011-D1RA03395F-s2114

RA-011-D1RA03395F-s2115

RA-011-D1RA03395F-s2116

RA-011-D1RA03395F-s2117

RA-011-D1RA03395F-s2118

RA-011-D1RA03395F-s2119

RA-011-D1RA03395F-s2120

RA-011-D1RA03395F-s2121

RA-011-D1RA03395F-s2122

RA-011-D1RA03395F-s2123

RA-011-D1RA03395F-s2124

RA-011-D1RA03395F-s2125

RA-011-D1RA03395F-s2126

RA-011-D1RA03395F-s2127

RA-011-D1RA03395F-s2128

RA-011-D1RA03395F-s2129

RA-011-D1RA03395F-s2130

RA-011-D1RA03395F-s2131

RA-011-D1RA03395F-s2132

RA-011-D1RA03395F-s2133

RA-011-D1RA03395F-s2134

RA-011-D1RA03395F-s2135

RA-011-D1RA03395F-s2136

RA-011-D1RA03395F-s2137

RA-011-D1RA03395F-s2138

RA-011-D1RA03395F-s2139

RA-011-D1RA03395F-s2140

RA-011-D1RA03395F-s2141

RA-011-D1RA03395F-s2142

RA-011-D1RA03395F-s2143

RA-011-D1RA03395F-s2144

RA-011-D1RA03395F-s2145

RA-011-D1RA03395F-s2146

RA-011-D1RA03395F-s2147

RA-011-D1RA03395F-s2148

RA-011-D1RA03395F-s2149

RA-011-D1RA03395F-s2150

RA-011-D1RA03395F-s2151

RA-011-D1RA03395F-s2152

RA-011-D1RA03395F-s2153

RA-011-D1RA03395F-s2154

RA-011-D1RA03395F-s2155

RA-011-D1RA03395F-s2156

RA-011-D1RA03395F-s2157

RA-011-D1RA03395F-s2158

RA-011-D1RA03395F-s2159

RA-011-D1RA03395F-s2160

RA-011-D1RA03395F-s2161

RA-011-D1RA03395F-s2162

RA-011-D1RA03395F-s2163

RA-011-D1RA03395F-s2164

RA-011-D1RA03395F-s2165

RA-011-D1RA03395F-s2166

RA-011-D1RA03395F-s2167

RA-011-D1RA03395F-s2168

RA-011-D1RA03395F-s2169

RA-011-D1RA03395F-s2170

RA-011-D1RA03395F-s2171

RA-011-D1RA03395F-s2172

RA-011-D1RA03395F-s2173

RA-011-D1RA03395F-s2174

RA-011-D1RA03395F-s2175

RA-011-D1RA03395F-s2176

RA-011-D1RA03395F-s2177

RA-011-D1RA03395F-s2178

RA-011-D1RA03395F-s2179

RA-011-D1RA03395F-s2180

RA-011-D1RA03395F-s2181

RA-011-D1RA03395F-s2182

RA-011-D1RA03395F-s2183

RA-011-D1RA03395F-s2184

RA-011-D1RA03395F-s2185

RA-011-D1RA03395F-s2186

RA-011-D1RA03395F-s2187

RA-011-D1RA03395F-s2188

RA-011-D1RA03395F-s2189

RA-011-D1RA03395F-s2190

RA-011-D1RA03395F-s2191

RA-011-D1RA03395F-s2192

RA-011-D1RA03395F-s2193

RA-011-D1RA03395F-s2194

RA-011-D1RA03395F-s2195

RA-011-D1RA03395F-s2196

RA-011-D1RA03395F-s2197

RA-011-D1RA03395F-s2198

RA-011-D1RA03395F-s2199

RA-011-D1RA03395F-s2200

RA-011-D1RA03395F-s2201

RA-011-D1RA03395F-s2202

RA-011-D1RA03395F-s2203

RA-011-D1RA03395F-s2204

RA-011-D1RA03395F-s2205

RA-011-D1RA03395F-s2206

RA-011-D1RA03395F-s2207

RA-011-D1RA03395F-s2208

RA-011-D1RA03395F-s2209

RA-011-D1RA03395F-s2210

RA-011-D1RA03395F-s2211

RA-011-D1RA03395F-s2212

RA-011-D1RA03395F-s2213

RA-011-D1RA03395F-s2214

RA-011-D1RA03395F-s2215

RA-011-D1RA03395F-s2216

RA-011-D1RA03395F-s2217

RA-011-D1RA03395F-s2218

RA-011-D1RA03395F-s2219

RA-011-D1RA03395F-s2220

RA-011-D1RA03395F-s2221

RA-011-D1RA03395F-s2222

RA-011-D1RA03395F-s2223

RA-011-D1RA03395F-s2224

RA-011-D1RA03395F-s2225

RA-011-D1RA03395F-s2226

RA-011-D1RA03395F-s2227

RA-011-D1RA03395F-s2228

RA-011-D1RA03395F-s2229

RA-011-D1RA03395F-s2230

RA-011-D1RA03395F-s2231

RA-011-D1RA03395F-s2232

RA-011-D1RA03395F-s2233

RA-011-D1RA03395F-s2234

RA-011-D1RA03395F-s2235

RA-011-D1RA03395F-s2236

RA-011-D1RA03395F-s2237

RA-011-D1RA03395F-s2238

RA-011-D1RA03395F-s2239

RA-011-D1RA03395F-s2240

RA-011-D1RA03395F-s2241

RA-011-D1RA03395F-s2242

RA-011-D1RA03395F-s2243

RA-011-D1RA03395F-s2244

RA-011-D1RA03395F-s2245

RA-011-D1RA03395F-s2246

RA-011-D1RA03395F-s2247

RA-011-D1RA03395F-s2248

RA-011-D1RA03395F-s2249

RA-011-D1RA03395F-s2250

RA-011-D1RA03395F-s2251

RA-011-D1RA03395F-s2252

RA-011-D1RA03395F-s2253

RA-011-D1RA03395F-s2254

RA-011-D1RA03395F-s2255

RA-011-D1RA03395F-s2256

RA-011-D1RA03395F-s2257

RA-011-D1RA03395F-s2258

RA-011-D1RA03395F-s2259

RA-011-D1RA03395F-s2260

RA-011-D1RA03395F-s2261

RA-011-D1RA03395F-s2262

RA-011-D1RA03395F-s2263

RA-011-D1RA03395F-s2264

RA-011-D1RA03395F-s2265

RA-011-D1RA03395F-s2266

RA-011-D1RA03395F-s2267

RA-011-D1RA03395F-s2268

RA-011-D1RA03395F-s2269

RA-011-D1RA03395F-s2270

RA-011-D1RA03395F-s2271

RA-011-D1RA03395F-s2272

RA-011-D1RA03395F-s2273

RA-011-D1RA03395F-s2274

RA-011-D1RA03395F-s2275

RA-011-D1RA03395F-s2276

RA-011-D1RA03395F-s2277

RA-011-D1RA03395F-s2278

RA-011-D1RA03395F-s2279

RA-011-D1RA03395F-s2280

RA-011-D1RA03395F-s2281

RA-011-D1RA03395F-s2282

RA-011-D1RA03395F-s2283

RA-011-D1RA03395F-s2284

RA-011-D1RA03395F-s2285

RA-011-D1RA03395F-s2286

RA-011-D1RA03395F-s2287

RA-011-D1RA03395F-s2288

RA-011-D1RA03395F-s2289

RA-011-D1RA03395F-s2290

RA-011-D1RA03395F-s2291

RA-011-D1RA03395F-s2292

RA-011-D1RA03395F-s2293

RA-011-D1RA03395F-s2294

RA-011-D1RA03395F-s2295

RA-011-D1RA03395F-s2296

RA-011-D1RA03395F-s2297

RA-011-D1RA03395F-s2298

RA-011-D1RA03395F-s2299

RA-011-D1RA03395F-s2300

RA-011-D1RA03395F-s2301

RA-011-D1RA03395F-s2302

RA-011-D1RA03395F-s2303

RA-011-D1RA03395F-s2304

RA-011-D1RA03395F-s2305

RA-011-D1RA03395F-s2306

RA-011-D1RA03395F-s2307

RA-011-D1RA03395F-s2308

RA-011-D1RA03395F-s2309

RA-011-D1RA03395F-s2310

RA-011-D1RA03395F-s2311

RA-011-D1RA03395F-s2312

RA-011-D1RA03395F-s2313

RA-011-D1RA03395F-s2314

RA-011-D1RA03395F-s2315

RA-011-D1RA03395F-s2316

RA-011-D1RA03395F-s2317

RA-011-D1RA03395F-s2318

RA-011-D1RA03395F-s2319

RA-011-D1RA03395F-s2320

RA-011-D1RA03395F-s2321

RA-011-D1RA03395F-s2322

RA-011-D1RA03395F-s2323

RA-011-D1RA03395F-s2324

RA-011-D1RA03395F-s2325

RA-011-D1RA03395F-s2326

RA-011-D1RA03395F-s2327

RA-011-D1RA03395F-s2328

RA-011-D1RA03395F-s2329

RA-011-D1RA03395F-s2330

RA-011-D1RA03395F-s2331

RA-011-D1RA03395F-s2332

RA-011-D1RA03395F-s2333

RA-011-D1RA03395F-s2334

RA-011-D1RA03395F-s2335

RA-011-D1RA03395F-s2336

RA-011-D1RA03395F-s2337

RA-011-D1RA03395F-s2338

RA-011-D1RA03395F-s2339

RA-011-D1RA03395F-s2340

RA-011-D1RA03395F-s2341

RA-011-D1RA03395F-s2342

RA-011-D1RA03395F-s2343

RA-011-D1RA03395F-s2344

RA-011-D1RA03395F-s2345

RA-011-D1RA03395F-s2346

RA-011-D1RA03395F-s2347

RA-011-D1RA03395F-s2348

RA-011-D1RA03395F-s2349

RA-011-D1RA03395F-s2350

RA-011-D1RA03395F-s2351

RA-011-D1RA03395F-s2352

RA-011-D1RA03395F-s2353

RA-011-D1RA03395F-s2354

RA-011-D1RA03395F-s2355

RA-011-D1RA03395F-s2356

RA-011-D1RA03395F-s2357

RA-011-D1RA03395F-s2358

RA-011-D1RA03395F-s2359

RA-011-D1RA03395F-s2360

RA-011-D1RA03395F-s2361

RA-011-D1RA03395F-s2362

RA-011-D1RA03395F-s2363

RA-011-D1RA03395F-s2364

RA-011-D1RA03395F-s2365

RA-011-D1RA03395F-s2366

RA-011-D1RA03395F-s2367

RA-011-D1RA03395F-s2368

RA-011-D1RA03395F-s2369

RA-011-D1RA03395F-s2370

RA-011-D1RA03395F-s2371

RA-011-D1RA03395F-s2372

RA-011-D1RA03395F-s2373

RA-011-D1RA03395F-s2374

RA-011-D1RA03395F-s2375

RA-011-D1RA03395F-s2376

RA-011-D1RA03395F-s2377

RA-011-D1RA03395F-s2378

RA-011-D1RA03395F-s2379

RA-011-D1RA03395F-s2380

RA-011-D1RA03395F-s2381

RA-011-D1RA03395F-s2382

RA-011-D1RA03395F-s2383

RA-011-D1RA03395F-s2384

RA-011-D1RA03395F-s2385

RA-011-D1RA03395F-s2386

RA-011-D1RA03395F-s2387

RA-011-D1RA03395F-s2388

RA-011-D1RA03395F-s2389

RA-011-D1RA03395F-s2390

RA-011-D1RA03395F-s2391

RA-011-D1RA03395F-s2392

RA-011-D1RA03395F-s2393

RA-011-D1RA03395F-s2394

RA-011-D1RA03395F-s2395

RA-011-D1RA03395F-s2396

RA-011-D1RA03395F-s2397

RA-011-D1RA03395F-s2398

RA-011-D1RA03395F-s2399

RA-011-D1RA03395F-s2400

RA-011-D1RA03395F-s2401

RA-011-D1RA03395F-s2402

RA-011-D1RA03395F-s2403

RA-011-D1RA03395F-s2404

RA-011-D1RA03395F-s2405

RA-011-D1RA03395F-s2406

RA-011-D1RA03395F-s2407

RA-011-D1RA03395F-s2408

RA-011-D1RA03395F-s2409

RA-011-D1RA03395F-s2410

RA-011-D1RA03395F-s2411

RA-011-D1RA03395F-s2412

RA-011-D1RA03395F-s2413

RA-011-D1RA03395F-s2414

RA-011-D1RA03395F-s2415

RA-011-D1RA03395F-s2416

RA-011-D1RA03395F-s2417

RA-011-D1RA03395F-s2418

RA-011-D1RA03395F-s2419

RA-011-D1RA03395F-s2420

RA-011-D1RA03395F-s2421

RA-011-D1RA03395F-s2422

RA-011-D1RA03395F-s2423

RA-011-D1RA03395F-s2424

RA-011-D1RA03395F-s2425

RA-011-D1RA03395F-s2426

RA-011-D1RA03395F-s2427

RA-011-D1RA03395F-s2428

RA-011-D1RA03395F-s2429

RA-011-D1RA03395F-s2430

RA-011-D1RA03395F-s2431

RA-011-D1RA03395F-s2432

RA-011-D1RA03395F-s2433

RA-011-D1RA03395F-s2434

RA-011-D1RA03395F-s2435

RA-011-D1RA03395F-s2436

RA-011-D1RA03395F-s2437

RA-011-D1RA03395F-s2438

RA-011-D1RA03395F-s2439

RA-011-D1RA03395F-s2440

RA-011-D1RA03395F-s2441

RA-011-D1RA03395F-s2442

RA-011-D1RA03395F-s2443

RA-011-D1RA03395F-s2444

RA-011-D1RA03395F-s2445

RA-011-D1RA03395F-s2446

RA-011-D1RA03395F-s2447

RA-011-D1RA03395F-s2448

RA-011-D1RA03395F-s2449

RA-011-D1RA03395F-s2450

RA-011-D1RA03395F-s2451

RA-011-D1RA03395F-s2452

RA-011-D1RA03395F-s2453

RA-011-D1RA03395F-s2454

RA-011-D1RA03395F-s2455

RA-011-D1RA03395F-s2456

RA-011-D1RA03395F-s2457

RA-011-D1RA03395F-s2458

RA-011-D1RA03395F-s2459

RA-011-D1RA03395F-s2460

RA-011-D1RA03395F-s2461

RA-011-D1RA03395F-s2462

RA-011-D1RA03395F-s2463

RA-011-D1RA03395F-s2464

RA-011-D1RA03395F-s2465

RA-011-D1RA03395F-s2466

RA-011-D1RA03395F-s2467

RA-011-D1RA03395F-s2468

RA-011-D1RA03395F-s2469

RA-011-D1RA03395F-s2470

RA-011-D1RA03395F-s2471

RA-011-D1RA03395F-s2472

RA-011-D1RA03395F-s2473

RA-011-D1RA03395F-s2474

RA-011-D1RA03395F-s2475

RA-011-D1RA03395F-s2476

RA-011-D1RA03395F-s2477

RA-011-D1RA03395F-s2478

RA-011-D1RA03395F-s2479

RA-011-D1RA03395F-s2480

RA-011-D1RA03395F-s2481

RA-011-D1RA03395F-s2482

RA-011-D1RA03395F-s2483

RA-011-D1RA03395F-s2484

RA-011-D1RA03395F-s2485

RA-011-D1RA03395F-s2486

RA-011-D1RA03395F-s2487

RA-011-D1RA03395F-s2488

RA-011-D1RA03395F-s2489

RA-011-D1RA03395F-s2490

RA-011-D1RA03395F-s2491

RA-011-D1RA03395F-s2492

RA-011-D1RA03395F-s2493

RA-011-D1RA03395F-s2494

RA-011-D1RA03395F-s2495

RA-011-D1RA03395F-s2496

RA-011-D1RA03395F-s2497

RA-011-D1RA03395F-s2498

RA-011-D1RA03395F-s2499

RA-011-D1RA03395F-s2500

RA-011-D1RA03395F-s2501

RA-011-D1RA03395F-s2502

RA-011-D1RA03395F-s2503

RA-011-D1RA03395F-s2504

RA-011-D1RA03395F-s2505

RA-011-D1RA03395F-s2506

RA-011-D1RA03395F-s2507

RA-011-D1RA03395F-s2508

RA-011-D1RA03395F-s2509

RA-011-D1RA03395F-s2510
